# Enhancers with cooperative Notch binding sites are more resistant to regulation by the Hairless co-repressor

**DOI:** 10.1371/journal.pgen.1009039

**Published:** 2021-09-24

**Authors:** Yi Kuang, Anna Pyo, Natanel Eafergan, Brittany Cain, Lisa M. Gutzwiller, Ofri Axelrod, Ellen K. Gagliani, Matthew T. Weirauch, Raphael Kopan, Rhett A. Kovall, David Sprinzak, Brian Gebelein

**Affiliations:** 1 Graduate Program in Molecular and Developmental Biology, Cincinnati Children’s Hospital Research Foundation, Cincinnati, Ohio, United States of America; 2 Department of Biomedical Engineering, University of Cincinnati, Cincinnati, Ohio, United States of America; 3 School of Neurobiology, Biochemistry and Biophysics, George S. Wise Faculty of Life Science, Tel Aviv University, Tel Aviv, Israel; 4 Division of Developmental Biology, Cincinnati Children’s Hospital Medical Center, Cincinnati, Ohio, United States of America; 5 Department of Molecular Genetics, Biochemistry and Microbiology, University of Cincinnati College of Medicine, Cincinnati, Ohio, United States of America; 6 Divisions of Biomedical Informatics and Developmental Biology, Center for Autoimmune Genomics and Etiology (CAGE), Cincinnati Children’s Hospital Medical Center, Cincinnati, Ohio, United States of America; 7 Department of Pediatrics, University of Cincinnati College of Medicine, Cincinnati, Ohio, United States of America; Dana-Farber Cancer Institute, XX

## Abstract

Notch signaling controls many developmental processes by regulating gene expression. Notch-dependent enhancers recruit activation complexes consisting of the Notch intracellular domain, the Cbf/Su(H)/Lag1 (CSL) transcription factor (TF), and the Mastermind co-factor via two types of DNA sites: monomeric CSL sites and cooperative dimer sites called Su(H) paired sites (SPS). Intriguingly, the CSL TF can also bind co-repressors to negatively regulate transcription via these same sites. Here, we tested how synthetic enhancers with monomeric CSL sites versus dimeric SPSs bind *Drosophila* Su(H) complexes *in vitro* and mediate transcriptional outcomes *in vivo*. Our findings reveal that while the Su(H)/Hairless co-repressor complex similarly binds SPS and CSL sites in an additive manner, the Notch activation complex binds SPSs, but not CSL sites, in a cooperative manner. Moreover, transgenic reporters with SPSs mediate stronger, more consistent transcription and are more resistant to increased Hairless co-repressor expression compared to reporters with the same number of CSL sites. These findings support a model in which SPS containing enhancers preferentially recruit cooperative Notch activation complexes over Hairless repression complexes to ensure consistent target gene activation.

## Author summary

Cell signaling provides a basic means of communication during development. Many signaling pathways, including the Notch pathway, convert extracellular signals into changes in gene expression via transcription factors that bind specific DNA sequences. Importantly, the Notch pathway transcription factor can either form activating complexes upon Notch activation to stimulate gene expression or repression complexes with co-repressors to inhibit gene expression. Prior studies showed that the Notch activation complex binds DNA as either an independent complex on monomer binding sites or as two cooperative complexes (dimer) on paired binding sites. In this study, we used synthetic biology to examine how these two types of DNA sites impact the binding of Notch activation versus repression complexes and the output of Notch target gene expression. Our studies reveal that unlike the Notch activation complex, the repression complex does not cooperatively bind dimer sites. Moreover, our findings support the model that the enhanced stability of the Notch activation complex on dimer sites makes target genes with dimer sites less sensitive to the repression complex than target genes with only monomer sites. Thus, our studies reveal how target genes with different binding sites differ in sensitivity to the ratio of Notch activation to repression complexes.

## Introduction

Notch signaling is a highly conserved cell-to-cell communication pathway that conveys information required for proper cellular decisions in many tissues and organs. During embryonic development, the Notch signaling pathway is used to specify distinct cell fates and thereby plays crucial roles during organogenesis including vasculogenesis [1], hematopoiesis [2], neurogenesis [3,4], and cardiac development [5–7]. Additionally, Notch regulates tissue homeostasis, including epidermal differentiation and maintenance [8], lymphocyte differentiation [9], muscle and bone regeneration [10–12], and angiogenesis [1]. Intriguingly, Notch regulates these diverse processes using a common molecular cascade that is initiated through a ligand (Delta/Serrate/Jagged)-receptor (Notch) interaction that triggers the cleavage and release of the Notch intracellular domain (NICD) into the cytoplasm. NICD subsequently translocates into the nucleus and forms a ternary complex with the Cbf1/Su(H)/Lag1 (CSL) transcription factor (which is also commonly called RBPJ in mammals) and the Mastermind (Mam) adapter protein. The NICD/CSL/Mam (NCM) complex recruits the p300 co-activator to activate the expression of Notch target genes required for proper cellular outcomes [13,14].

Since NICD and Mam do not directly bind DNA, the targeting of the NCM complex to specific genomic loci is determined by the CSL transcription factor (TF). Both *in vitro* and *in vivo* DNA binding assays show that the CSL TFs from *C elegans* [15], *Drosophila* [16], and vertebrates [17–20] bind highly similar DNA sequences (i.e. ^T^/_C_GTG^G^/_A_GAA), and its interactions with Notch and Mam were found to not significantly alter CSL DNA binding specificity in unbiased protein binding microarray studies [21]. Interestingly, studies in flies and mammals found that a subset of Notch target genes contain enhancers with two binding sites spaced 15 to 17bp apart and oriented in a head-to-head manner [22–24]. Subsequent biochemical and structural studies revealed that such sites, which have been named Su(H) paired sites or sequence paired sites (SPSs), mediate cooperative NCM binding due to dimerization between two adjacent NICD molecules [25,26].

SPSs are present in a substantial fraction of Notch-dependent enhancers in the genome. In human CUTLL1 T-cell acute lymphoblastic leukemia (T-ALL) cell line, 36% (38 of 107) of the high confidence Notch targets are dimer-dependent [24], and SPS-containing enhancers were found to be crucial for the maturation of both normal T-cells and the progression of T-ALL [27,28]. A genome-wide NICD complementation assay revealed that mouse mK4 kidney cells have as many as 2,500 Notch dimer-dependent loci [29]. Moreover, reporter assays and/or RT-PCR assays have tested the function of a small subset of these SPS containing enhancers and found that SPSs are typically required for optimal transcriptional responses [25–27,29]. A recent study also found that while mice with Notch1 and Notch2 point mutations that abolish cooperative binding to SPSs develop normally under ideal laboratory conditions, stressing the animals either genetically or with parasites can result in profound defects in gastrointestinal, cardiovascular, and immune systems [30]. Collectively, these studies revealed that many Notch-dependent target genes contain SPSs, and that the regulation of dimer-dependent Notch target genes contributes to animal development and homeostasis.

In addition to mediating Notch induced gene expression, the CSL TF can use the same DNA binding sites to repress transcription by recruiting co-repressor proteins. The *Drosophila* CSL transcription factor Su(H) binds to the Hairless (H) protein, which recruits either the Groucho (Gro) or the C-terminal binding protein (Ctbp) co-repressors [31–33]. The mammalian CSL transcription factor RBPJ interacts with several transcriptional repressors including the SHARP/Mint protein and Fhl1C/KyoT2 [34]. Once bound to DNA, these co-repressor complexes recruit additional proteins that can mediate transcriptional repression by modifying chromatin. Importantly, recent structural analysis of the fly and mammalian co-repressors bound to CSL and DNA showed that co-repressors interact with the CSL TF via the same binding domain as the NICD/Mam co-activators [35–37]. Thus, the co-repressors and co-activators are thought to bind the CSL transcription factor in a mutually exclusive manner. Moreover, genetic studies revealed that the ratio of the co-activator to co-repressor complex is critical for proper Notch-mediated cellular decisions, as lowering the gene dose of the *Hairless* co-repressor can suppress *Notch* haploinsufficiency phenotypes in *Drosophila* [38–41]. Lastly, recent live imaging studies showed that stimulating Notch signaling in cells results in increased binding of both Su(H) and the Hairless co-repressor to the well-known Enhancer of split (E(spl)) Notch target locus [42]. In total, these data support a model whereby the Notch activation complex directly competes for genomic binding sites with the CSL/co-repressor complex to regulate target gene expression.

Recent studies have begun to focus on defining whether Notch regulated enhancers with SPSs convey distinct transcriptional responses from CSL monomeric sites. For example, the *E(spl)* genes, many of which contain SPSs, were found to be among the first to respond after a short pulse of Notch activation in *Drosophila* DmD8 cells [43], consistent with SPS-containing enhancers responding quickly to low levels of Notch activation. However, subsequent live imaging studies comparing the activities of enhancers with SPS *versus* CSL sites revealed that the presence of SPSs did not significantly alter the sensitivity to NICD but instead enhanced transcriptional burst size [44]. It should be noted, however, that these studies have largely focused on how the Notch activation complex cooperatively binds to and impacts the regulation of SPS containing enhancers, whereas less is known about whether and how the SPS versus monomeric CSL sites differentially recruit the CSL/co-repressor complexes. Thus, it remains unclear how the levels of the co-repressors impact Notch regulated enhancers that contain cooperative SPS sites versus independent CSL sites.

Comparing Notch-mediated transcriptional responses of endogenous enhancers with SPS and CSL sites is complicated by several inherent properties of endogenous enhancers. First, most Notch-regulated SPS-containing enhancers also have variable numbers of independent monomer CSL sites. Second, endogenous enhancers contain distinct combinations of additional TF binding sites that can significantly alter transcriptional output. Third, each endogenous enhancer is embedded in its own unique chromosomal environment, which can further impact the ability of Notch transcription complexes to regulate gene expression. In this study, we circumvented these confounders by integrating transgenic reporters containing either synthetic SPS or CSL enhancers to focus our investigation on how the architecture of Su(H) binding sites impacts Notch transcriptional output in *Drosophila*. We complemented these studies using *in vitro* DNA binding assays to assess how SPS versus CSL sites impact the binding of the NCM versus CSL/co-repressor complexes. Altogether, our data reveal that Notch regulated enhancers containing cooperative SPSs are more resistant to the Hairless co-repressor protein than enhancers with independent CSL sites. Integrating this study with previously published data provides new insights into how DNA binding site architecture affects transcriptional output by both modulating transcriptional dynamics and by competing with the co-repressors that limit transcriptional activation.

## Results

### Activating but not repressing Su(H) complexes cooperatively bind SPS sites in vitro

**Fig 1:**
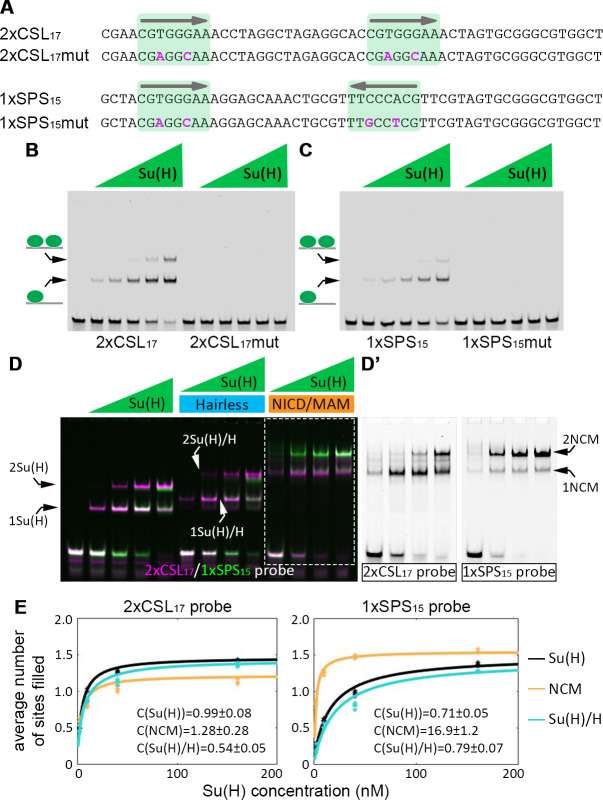
The Notch-CSL-Mastermind (NCM) complex binds the SPS sequence cooperatively *in vitro*. **A**. Sequences of the 2xCSL_17_ and 1xSPS_15_ probes, which both contain two consensus Su(H) binding sites (CGTGGGAA, highlighted in green) that only differ in orientation and spacing. The specific mutations introduced into the Su(H) binding sites are noted in magenta text. **B-C**. EMSAs reveal binding of purified *Drosophila* Su(H) to the wild type, but not the mutated, 2xCSL_17_ (B) and 1xSPS_15_ (C) probes. Su(H) concentration increases from 0.94nM to 15nM in 2-fold steps. **D**. EMSA data reveals binding of the indicated purified *Drosophila* proteins on 2xCSL_17_ (magenta) or 1xSPS_15_ (green) probes. Note, Su(H) alone and the Su(H)/H co-repressor complex bind the 2xCSL_17_ and 1xSPS_15_ probes in a largely additive manner. In contrast, the NCM co-activator complex (arrows at right) binds the 1xSPS_15_ but not the 2xCSL_17_ probe cooperatively. Su(H) concentration increases from 2.5 to 160 nM in 4-fold steps and 2 μM Hairless or NICD/Mam was used in indicated lanes. Note, we separated the two colors for the NCM activating complex in grayscale in D’ and show the entire gel in grayscale in **S1A** and **S1B Fig**. **E**. Average number of sites filled with increasing amounts of Su(H) in reactions of Su(H) alone (black), Su(H) with co-activators (orange) and Su(H) with the Hairless (H) co-repressor (blue). The average number of sites filled is defined as $\bar{n}=\frac{I_{1}}{I_{0}+I_{1}+I_{2}}+2\frac{I_{2}}{I_{0}+I_{1}+I_{2}}$, where *I*_0_, *I*_1_ and *I*_2_ are the extracted band values for the 0, 1, and 2 TFs bound to the probe. Each reaction was repeated four times and the dots represent data from each individual experiment. Lines represent fitted data (see Materials and Methods). Extracted cooperativity factors-C are as indicated.

To study the ability of Notch monomer (CSL) and Notch dimer (SPS) sites to bind activating (NICD/CSL/Mam, NCM) and repressing (CSL/Hairless) complexes that regulate gene expression in *Drosophila*, we created synthetic CSL and SPS enhancers for both electrophoretic mobility shift assays (EMSAs) and *in vivo* transgenic reporter assays. To design each synthetic sequence, we anchored two consensus Su(H) sites (CGTGGGAA) as defined by prior studies [45] a suitable distance apart (15-17bps) in either a head-to-head (SPS) configuration to promote cooperative NCM binding or a head-to-tail configuration to promote independent NCM binding (**Fig 1A**). The intervening sequences were subsequently selected to limit the inclusion of other known transcription factor binding sites (TFBSs) by randomly generating thousands of sequence variants and scoring each using a TF binding motif database (CIS-BP, http://cisbp.ccbr.utoronto.ca) [46]. Using this approach, we selected a 2xCSL sequence with a 17bp spacer (2xCSL_17_) and a 1xSPS sequence with a 15bp spacer (1xSPS_15_, **Fig 1A**), and we examined the specificity of the engineered 2xCSL_17_ and 1xSPS_15_ DNA sequences for Su(H) binding (note, 2xCSL_17_ and 1xSPS_15_ have the same number of Su(H) binding sites) using two *in vitro* DNA binding assays: First, we found that purified Su(H) protein binds DNA probes containing the 2xCSL_17_ and 1xSPS_15_ sequences, but not probes with point mutations in the Su(H) sites (**Fig 1A, 1B and 1C**). Second, we tested how the orientation of the sites affects Su(H) DNA binding in the presence of NICD and Mam (i.e. the NCM activating complex) or the Hairless (H) co-repressor. For this experiment, we used purified proteins that include the NICD (aa 1763–2142), Mam (aa 87–307) and Hairless (aa 232–358) domains required to form stable complexes with Su(H), and we directly compared the binding of each TF complex using differentially labeled 2xCSL_17_ (700nm wavelength, pseudo-colored magenta) and 1xSPS_15_ (800nm wavelength, pseudo-colored green) probes in the same reaction (**Fig 1D**). Importantly, we found that like Su(H) alone, the Su(H)/H complex bound both the 2xCSL_17_ and 1xSPS_15_ probe in an additive manner (**Figs 1D**, **S1A** and **S1B**). In sharp contrast, the NCM complex preferentially formed larger TF complexes, consistent with filling both sites of the 1xSPS_15_ probe, compared to the sequential binding to 2xCSL_17_ (**Fig 1D** and **1D’**). Thus, unlike the NCM co-activator complex, the Su(H)/H repression complex does not bind to SPS sites in a cooperative manner.

To obtain a measure of the cooperativity induced by NCM binding to the 1xSPS_15_ vs 2xCSL_17_ probes, we quantitatively analyzed the band intensities in the EMSA gels and fitted the extracted values to a 2-site equilibrium binding model (**Fig 1E**). The model takes into account cooperative binding by assuming that the dissociation constant associated with the second binding, *K*_*d*2_, is smaller by a cooperativity factor, C, with respect to the dissociation constant associated with the first binding, *K*_*d*1_, such that *K*_*d*2_ = *K*_*d*1_/*C*. A cooperativity factor higher than 1 corresponds to positive cooperative binding. A cooperativity factor close to 1 or smaller than 1 corresponds to non-cooperative binding and negative cooperative binding (i.e. steric hindrance), respectively. Fitting the band intensities from the EMSA experiments allowed the extraction of the cooperativity factor for each complex and each probe **(S1 Fig)**. Other than the NCM complex on SPS, all other experimental conditions exhibited cooperativity factors close to 1, indicating a non-cooperative binding process. In contrast, the NCM complex had a cooperativity factor of 16.9±1.2 on the SPS probe, clearly showing a strong cooperative binding (higher than 16-fold).

**Fig 2:**
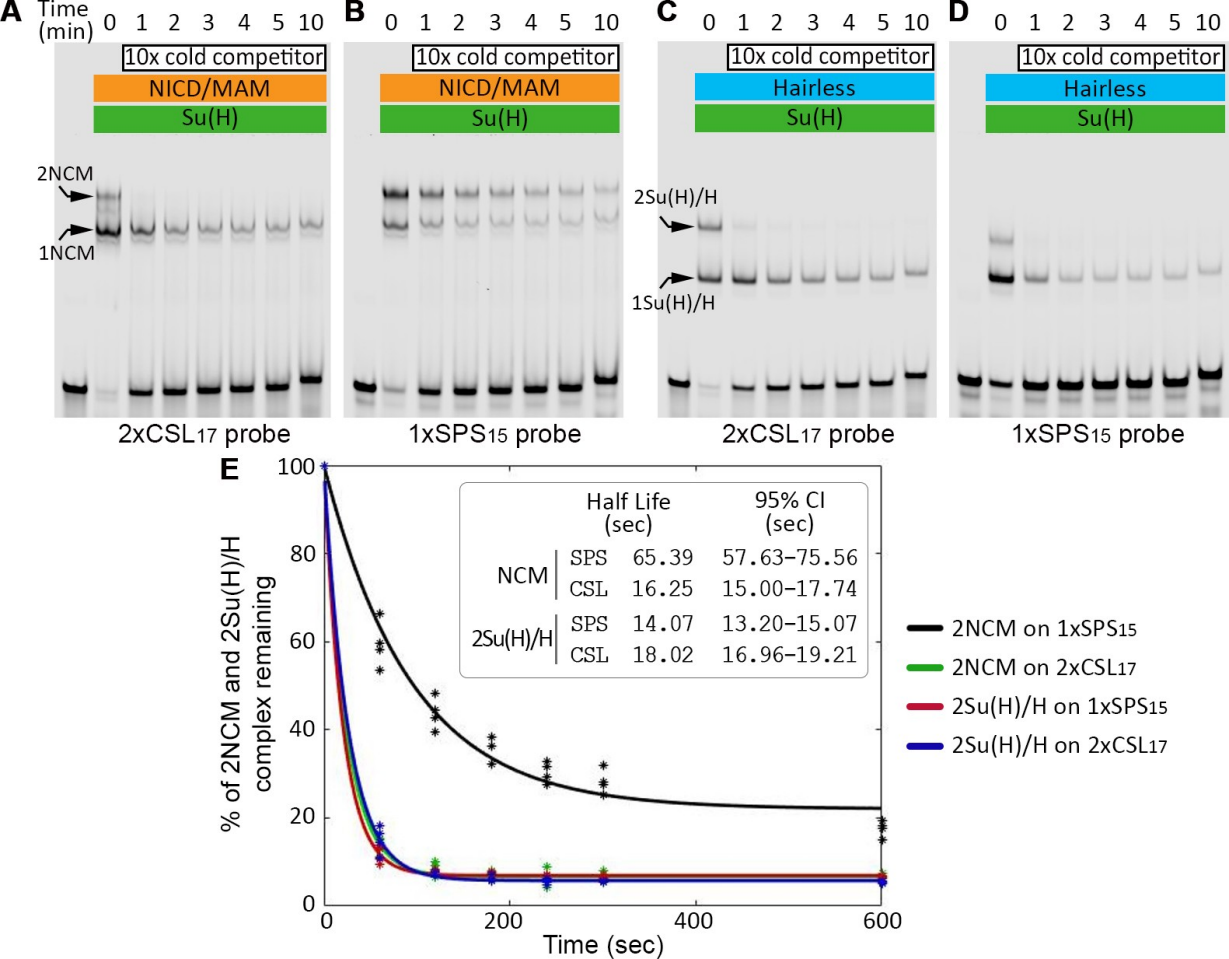
Dimeric NCM complexes dissociate more slowly from the SPS probe than from CSL probe. **A-D.** EMSAs reveal binding of either the NCM co-activator complex to the 2xCSL_17_ (A) and 1xSPS_15_ (B) probes or the Su(H)/H co-repressor complex to the 2xCSL_17_ (C) and 1xSPS_15_ (D) probes after incubating with 10x unlabeled 2xCSL_17_ competitor probe for the indicated time periods. 3.5nM labeled probe, 80nM Su(H), and 2 μM of either Hairless or NICD/Mam was used in the indicated lanes. 35nM unlabeled 2xCSL_17_ probe was used as the cold competitor. **E.** Percentage of 2xCSL_17_ or 1xSPS_15_ probes bound by 2NCM or 2Su(H)/H complexes over time in the presence of cold competitor probes. Each reaction was repeated four times and the asterisks represent data from each individual experiment. Solid lines represent a fit to decaying exponents (see Materials and Methods). Extrapolated half-lives for each complex and 95% confidence intervals are indicated.

Cooperative binding of the 2NCM complexes on the 1xSPS_15_ probe most likely results in a slower off-rate on SPSs than CSL sites. To measure the dissociation rates for the Su(H) complexes on each type of probe, we performed a series of temporal cold competitor experiments. First, we established the specificity of competition using unlabeled 2xCSL_17_, 1xSPS_15_, and 2xCSL_17_-mutant sequences and found that adding either 2xCSL_17_ or 1xSPS_15_, but not the 2xCSL17-mutant sequence, effectively competed for the Su(H) TF (**S2 Fig**). Next, we performed a series of temporal competition assays by first adding either NICD/Su(H)/Mam (NCM) or Su(H)/H (co-R) to the labeled 2xCSL_17_ or 1xSPS_15_ probes and then adding 10x fold excess of the unlabeled 2xCSL_17_ probe for different lengths of time (see Materials and Methods for details). Importantly, while a 10-fold excess of cold 2xCSL_17_ competitor rapidly depleted the 2NCM band bound to the labeled 2xCSL_17_ probe, the 2NCM band was depleted much more slowly when bound to the labeled 1xSPS_15_ probe (**Fig 2A** and **2B**). In sharp contrast, the 2Su(H)/H co-repressor bound to either the 2xCSL_17_ or 1xSPS_15_ probes was similarly depleted by 10x cold competitor (**Fig 2C** and **2D**). By repeating this experiment in quadruplicates, we estimated the observed half-lives of the 2NCM and 2Su(H)/H bands on each probe and found that the 2NCM half-life from the 1xSPS_15_ probe (black line, **Fig 2E**) was significantly longer than when the 2NCM is bound to the 2xCSL_17_ probe (green line, **Fig 2E**). Importantly, the 2Su(H)/H co-repressor half-life on both the 1xSPS_15_ (red line, **Fig 2E**) and the 2xCSL_17_ probes (blue line, **Fig 2E**) was similar to each other as well as to the half-life of 2NCM bound to the 2xCSL_17_ probe (green line, **Fig 2E**). However, the calculated half-lives should be viewed as estimates, as EMSAs do not provide sufficient temporal resolution to obtain accurate measurements given how fast the non-cooperative 2NCM/2xCSL_17_, 2Su(H)/H/2xCSL_17_, and 2Su(H)/H/1xSPS_15_ complexes dissociate from each probe. Our data nonetheless clearly show that while the independent sites in the 2xCSL_17_ probe mediate similar DNA binding patterns and kinetics for both the co-activator and co-repressor complexes, the 1xSPS_15_ sites form more stable cooperative NCM activation complexes relative to the binding of the less stable Su(H)/co-repressor complex.

### Generation of synthetic SPS and CSL reporters to study Notch-mediated gene regulation in Drosophila

To determine if enhancers with Notch dimer (SPS) vs monomer (CSL) binding sites mediate similar or distinct transcriptional outputs *in vivo*, we generated a series of synthetic transgenic reporters to test in *Drosophila*. A fundamental challenge in using such an *in vivo* synthetic approach is to isolate the Su(H) (the *Drosophila* CSL TF) binding sites from other transcriptional inputs that can alter gene expression output. As described above and in the Materials and Methods section, we used a random sequence generator followed by analysis with the CIS-BP database [46] to select flanking sequences in the 1xSPS_15_ and 2xCSL_17_ enhancers that limit the inclusion of additional known TFBS motifs. However, it should be noted that it is not possible to eliminate all other potential TF inputs for two reasons: First, we lack knowledge of binding motifs for every *Drosophila* TF. Second, it is difficult to include consensus Su(H) sites that do not also encode additional, partially overlapping TF motifs for other factors (see **S3A** and **S3B Fig** for a list of the other potential TFBS motifs found in the 2xCSL_17_ and 1xSPS_15_ sequences). Hence, in addition to making transgenic reporter lines with equal numbers of the selected CSL_17_ (*12xCSL*_*17*_*-lacZ*) and SPS_15_ (*6xSPS*_*15*_*-lacZ*) sites, we generated reporters with point mutations in each Su(H) site (*12xCSL*_*17*_*mut-lacZ* and *6xSPS*_*15*_*mut-lacZ*), while otherwise having the same respective flanking sequences (see **Fig 1A, 1B** and **1C** for mutant sequences and data showing loss of Su(H) DNA binding). Lastly, since our experimental approach selected distinct flanking sequences for the *2xCSL*_*17*_ and *1xSPS*_*15*_ enhancers, we generated two additional reporters in which one of the Su(H) sites was “flipped” (i.e. reverse-complementation of the core 8bp Su(H) site) to convert the 2xCSL_17_ into a 1xSPS_17_ sequence and the 1xSPS_15_ into a 2xCSL_15_ sequence (**S4A Fig**). EMSA analysis revealed that purified Su(H) and Su(H)/H proteins bound both the new 1xSPS_17_ and 2xCSL_15_ probes in the expected additive manner, whereas the NCM complex bound the 1xSPS_17_ but not the 2xCSL_15_ in a cooperative manner (**S4B** and **S4C Fig)**. To assess if flipping the Su(H) site in each construct created new, unintended TFBS motifs, we used the SNP analysis function in CIS-BP [46] to directly compare the 2xCSL_17_ with the 1xSPS_17_ sequence and the 1xSPS_15_ with the 2xCSL_15_ sequence. This analysis revealed that the 1xSPS_17_ sequence had an additional 9 predicted TFBSs relative to the original 2xCSL_17_ sequence and the 2xCSL_15_ sequence simultaneously created 3 new sites while also resulting in the loss of 2 predicted TFBS motifs that were in the original 1xSPS_15_ sequence (**S3C Fig**). These predictive computational analyses highlight the difficulty in designing enhancer sequences that are exclusively regulated by a specific transcription input.

**Fig 3:**
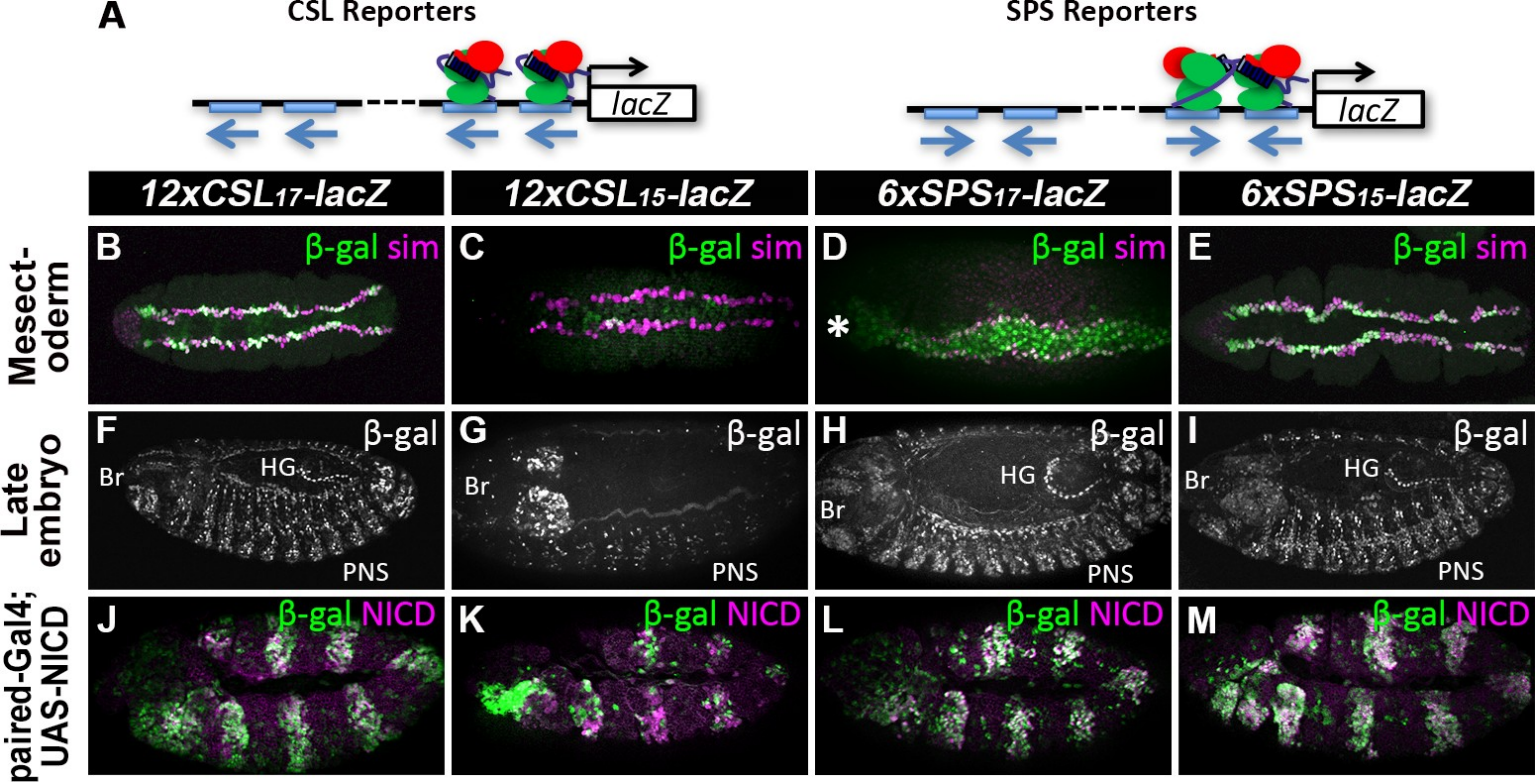
CSL and SPS reporters are expressed in multiple embryonic *Notch*-dependent tissues. **A**. Schematics of *CSL-lacZ* and *SPS-lacZ* reporter constructs with the orientation of each Su(H) binding site highlighted. **B-E**. Ventral view of stage 5 *Drosophila* embryos with the indicated reporters immunostained for β-gal (green) and Sim (magenta) revealed expression in the mesectoderm. Note, the relatively weak expression of the *12xCSL*_*15*_*-lacZ* reporter in the Sim-positive mesectoderm (C) and the asterisk denotes that the *6xSPS*_*17*_*-lacZ* reporter (D) has ectopic activity in the mesoderm. **F-I**. Lateral view of stage 15 *Drosophila* embryos with indicated reporters immunostained for β-gal revealed expression in expected Notch-dependent tissues including the embryonic brain (Br), cells associated with or within the peripheral nervous system (PNS), and the hindgut (HG). Note, the stability of the β-gal protein can reveal both current and prior transcriptional activity (i.e. serves as a short term marker), especially in cell types that receive a short pulse of Notch signaling such as during PNS development. In these embryos, the *12xCSL*_*17*_*-lacZ* and *6xSPS*_*15*_*-lacZ* reporters were inserted into ZH-86Fb locus, and *12xCSL*_*15*_*-lacZ* and *6xSPS*_*17*_*-lacZ* reporters were inserted into ZH-51C locus. Similar results were observed for the *12xCSL*_*17*_*-lacZ* and *6xSPS*_*15*_*-lacZ* reporters inserted into ZH-51C locus (see **S6 Fig**). **J**-**M**. Stage 11 *Drosophila* embryos containing the indicated reporters and *paired-Gal4>UAS-NICD* activation were immunostained for β-gal (green) and NICD (magenta). Note, all reporters were strongly activated by ectopic NICD (magenta stripes in embryos), except *12xCSL*_*15*_*-lacZ* was only sporadically expressed in the *PrdG4*-positive stripe. In this experiment, the *12xCSL*_*17*_*-lacZ*, *12xCSL*_*15*_*-lacZ*, *6xSPS*_*15*_*-lacZ*, and *6xSPS*_*17*_*-lacZ* reporters were all inserted into ZH-51C locus.

**Fig 4:**
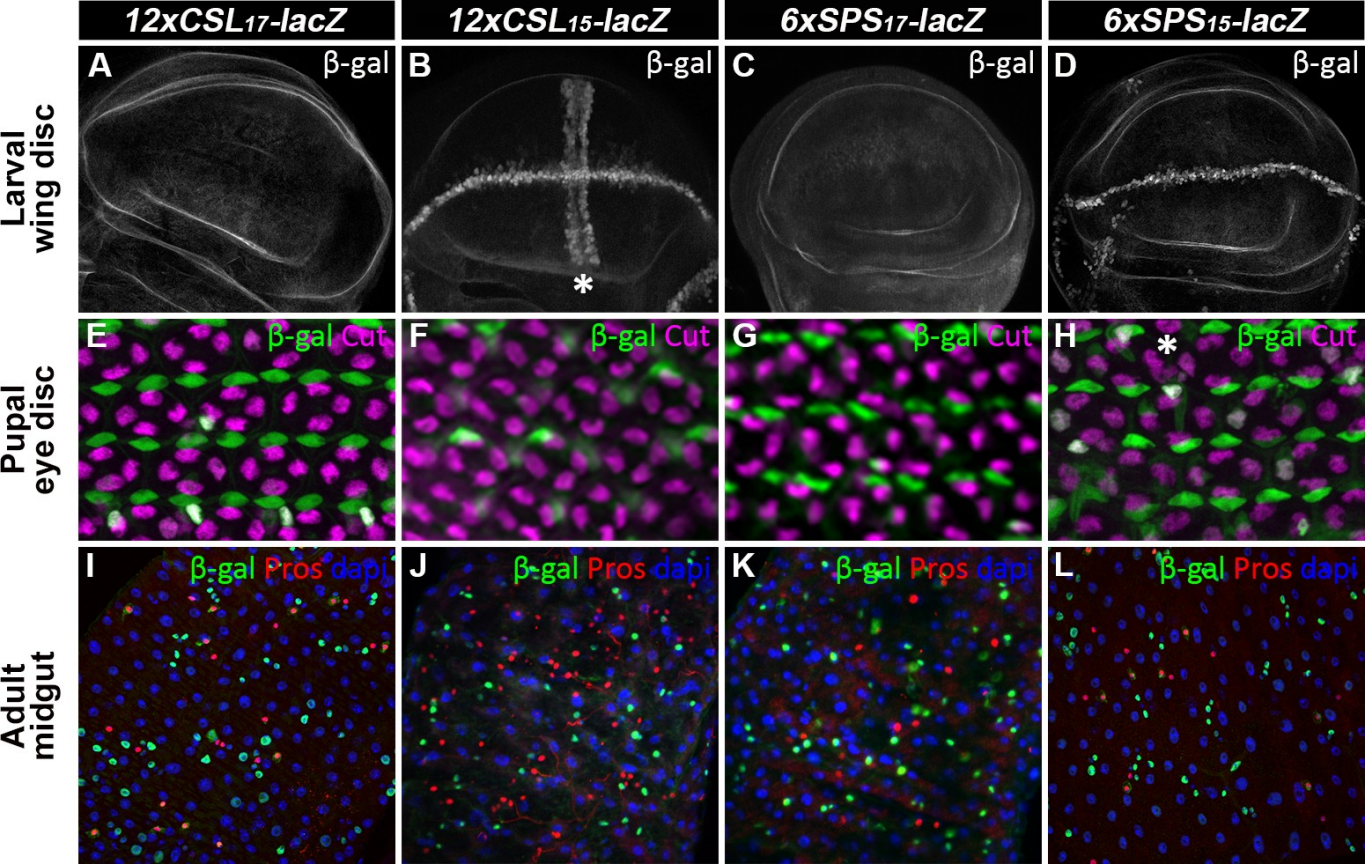
Expression analysis of the CSL and SPS reporters in larval, pupal, and adult tissues reveals the influence of flanking sequences on tissue-specific reporter activity. **A-D**. Larval wing discs with indicated Notch reporter vectors immunostained for β-gal revealed that only the CSL and SPS reporters with the 15bp flanking sequences were expressed in the D-V boundary (wing margin) cells (B,D). Note, that the *12xCSL*_*15*_*-lacZ* reporter was also expressed in wing pouch cells perpendicular to the wing margin (marked by * in B). **E-H**. Pupal eye discs with indicated reporters immunostained for β-gal (green) and cut (magenta), which marks the cone cells, revealed an expression pattern consistent with reporter activity in the primary pigment cells. * marks a cell missing reporter activity. **I-L**. Adult intestinal midgut cells with indicated reporters immunostained for β-gal (green), Pros (red), which is a marker of enteroendocrine cells, and counterstained with DAPI revealed an expression pattern in the smaller nuclei of the midgut, consistent with high Notch activity in the EB cells. The *12xCSL*_*17*_*-lacZ* and *6xSPS*_*15*_*-lacZ* reporters were inserted into ZH-86Fb locus, and *12xCSL*_*15*_*-lacZ* and *6xSPS*_*17*_*-lacZ* reporters were inserted into ZH-51C locus.

Next, we assessed the activity of these six reporters (*12xCSL*_*17*_*-lacZ*; *12xCSL*_*17*_*mut-lacZ*; *12xCSL*_*15*_*-lacZ*; *6xSPS*_*15*_*-lacZ*; *6xSPS*_*15*_*mut-lacZ*; and *6xSPS*_*17*_*-lacZ*) in a variety of *Drosophila* tissues by generating transgenic fly lines in which each was inserted into consistent chromosomal loci (51C and/or 86Fb) using *ϕ*C31 mediated recombination [47]. Importantly, expression analysis in *Drosophila* tissues revealed that the wild type CSL and SPS reporters, but not the mutant reporters (**S5 Fig**), activated qualitatively similar β-gal expression patterns in many Notch-dependent cell types (**Fig 3** and **Fig 4**). For example, the designed and selected *12xCSL*_*17*_*-lacZ* and *6xSPS*_*15*_*-lacZ* transgenes induced qualitatively similar reporter expression patterns in the embryonic mesectoderm as marked by the Single-minded (Sim) protein in the early embryo (**Fig 3B** and **3E** and [48]). In addition, we found that these two reporters were also expressed in many different cells of older embryos including in the brain [49], in cells within and closely associated with the peripheral nervous system [50,51], and in the hindgut dorsal-ventral boundary cells in older embryos (**Fig 3F** and **3I**, [52]). The *6xSPS*_*17*_*-lacZ* also had similar activity in the mesectoderm (**Fig 3D**) and in the nervous system and hindgut boundary cells of later embryos (**Fig 3H**), although this transgene was also ectopically expressed in the mesoderm of early embryos (the mesoderm is located between the two mesectoderm stripes, marked by an asterisk in **Fig 3D**). In contrast, the *12xCSL*_*15*_*-lacZ* transgene was much less active in the embryo as evidenced by the sporadic β-gal expression in the mesectoderm in the early embryo (**Fig 3C**) and by many fewer cells outside of the brain region activating *12xCSL*_*15*_*-lacZ* in older embryos (**Fig 3G**). To further test the decreased sensitivity of this *12xCSL*_*15*_*-lacZ* reporter to high levels of Notch signaling in the embryo, we used *paired-Gal4* (*prdG4*) to activate a *UAS-NICD* transgene in every other parasegment and found that while *12xCSL*_*17*_*-lacZ*, *6xSPS*_*15*_*-lacZ*, and *6xSPS*_*17*_*-lacZ* were each strongly activated by ectopic NICD, *12xCSL*_*15*_*-lacZ* was again activated in a sporadic manner (**Fig 3J, 3K, 3L** and **3M**). Thus, the designed and selected CSL_17_ and SPS_15_ reporters activated qualitatively similar expression patterns in the embryo, whereas those engineered with the “flipped” Su(H) sites either had ectopic activity (SPS_17_) or were active in fewer cells in the embryo (CSL_15_), potentially due to the creation of new transcription factor binding sites by inverting a single Su(H) site in each sequence (**S3C Fig**).

Next, we analyzed the activity of the synthetic reporters in larval imaginal discs, the pupal eye disc, and the adult midgut (**Fig 4**). Intriguingly, these comparative studies revealed the difficulty in designing enhancers that consistently respond to Notch signaling in all known Notch-dependent tissues, and these differences in tissue-specific activity were not due to the chromosomal integration site as the same reporters inserted into both 51C and 86Fb behaved in a similar manner (**S6 Fig**). For example, while all four CSL and SPS reporters similarly activated expression in the enteroblast cells of the adult midgut (**Fig 4I, 4J, 4K** and **4L**) [53,54], only the CSL and SPS reporters with the 15bp flanking sequences worked in the larval imaginal disc cells such as the wing margin cells (**Fig 4A, 4B, 4C** and **4D**), the leg joint boundary cells (**S7B, S7C, S7D** and **S7E Fig**), and in differentiating cells of the larval eye (**S7F, S7G, S7H** and **S7I Fig**) [55]. Moreover, the *12xCSL*_*15*_*-lacZ* reporter, but not the *6xSPS*_*15*_*-lacZ* reporter, drove an additional stripe of activity in the wing pouch that is perpendicular to the wing margin cells (see asterisk in **Fig 4B**), possibly due to the creation of a potential Scalloped (Sd) binding site [56] by flipping the Su(H) in the *6xSPS*_*15*_*-lacZ* (see **S3C Fig**). In sharp contrast the CSL and SPS reporters with the 17bp flanking sequences were not expressed well in any of the larval imaginal discs (**S7B, S7D, S7F** and **S7H Fig**). Intriguingly, however, these same Notch reporters worked very well and gave largely expected expression patterns in both the embryo (**Fig 3**) and the adult midgut (**Fig 4I, 4J, 4K** and **4L**). Moreover, comparative studies in the pupal eye disc revealed that the CSL and SPS reporters with the 17bp flanking sequences activated more consistent expression patterns in the primary pigment cells of the pupal eye [57,58] than the CSL and SPS reporters with 15bp flanking sequences (**Fig 4E, 4F, 4G** and **4H**). Taken together, these data show that while the designed synthetic *12xCSL*_*17*_*-lacZ* and *6xSPS*_*15*_*-lacZ* reporters behave as expected in the *Drosophila* embryo, we found that both the adjacent flanking sequences and flipping a single Su(H) site in each CSL and SPS construct can impact reporter output in a tissue-specific manner. These findings highlight the difficulty in designing universal Notch reporter genes that both work in all known Notch-dependent cell types and are not potentially influenced by additional TF inputs.

### SPS-lacZ reporters exhibit more consistent and stronger responses than CSL-lacZ reporters in the mesectoderm

**Fig 5:**
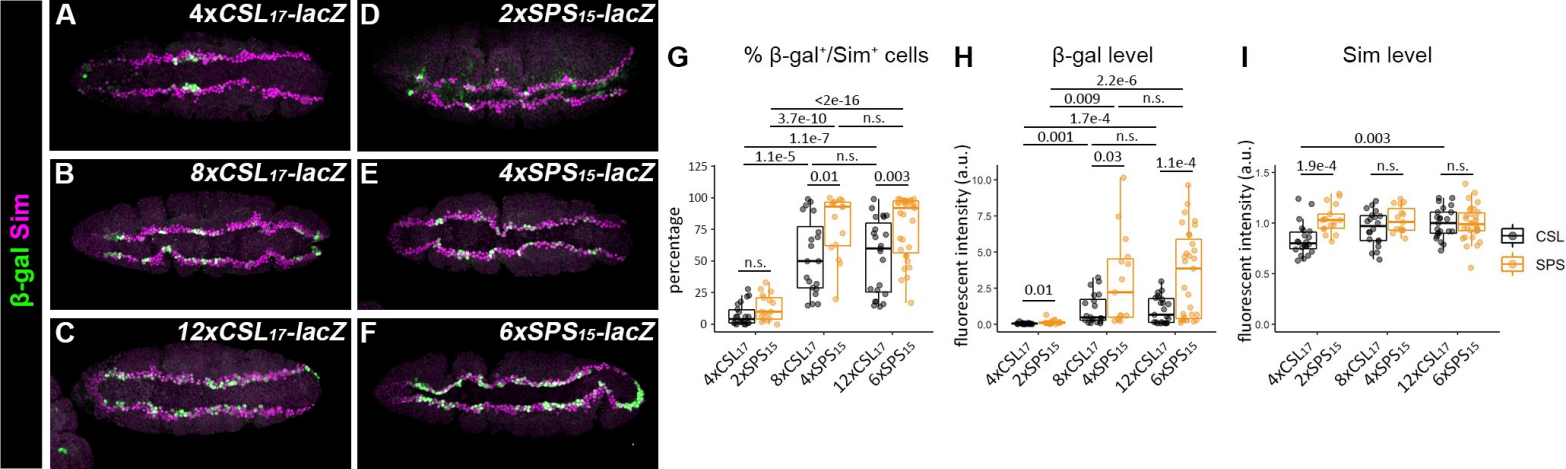
Cooperative binding sites enhance Notch transcriptional activity in the *Drosophila* mesectoderm. **A-F**. Ventral views of stage 5 *Drosophila* embryos carrying either the *4xCSL*_*17*_*-lacZ* (A), *8xCSL*_*17*_*-lacZ* (B), *12xCSL*_*17*_*-lacZ* (C), *2xSPS*_*15*_*-lacZ* (D), *4xSPS*_*15*_*-lacZ*, (E) or *6xSPS*_*15*_*-lacZ* (F) immunostained for β-gal (green) and Sim (magenta), which is a marker of mesectoderm cells. Each transgenic reporter was inserted into the ZH-86Fb locus. **G-I**. Quantification of the percentage of mesectoderm cells (Sim-positive cells) that activate β-gal (G), the mean β-gal protein levels (H) and the mean Sim protein levels (I) in flies containing the indicated reporters. Each dot represents the measurements from an individual embryo. Sample sizes (n) were 22 for *4xCSL*_*17*_, 17 for *2xSPS*_*15*_, 20 for *8xCSL*_*17*_, 15 for *4xSPS*_*15*_, 23 for *12xCSL*_*17*_, and 31 for *6xSPS*_*15*_. Box plots show the median, interquartile range, and 1.5 times interquartile range. One-way ANOVA with post-hoc Tukey HSD for equal variance or post-hoc Dunnett’s T3 for unequal variance were used to test significance. n.s. not significant.

The similar qualitative behaviors of the *12xCSL*_*17*_*-lacZ* and *6xSPS*_*15*_*-lacZ* in the *Drosophila* embryo provides an opportunity to perform a direct quantitative comparison between Notch monomer vs dimer enhancers using reporters integrated into a consistent locus (86Fb). Of the Notch active tissues in the embryo, mesectoderm specification provides an ideal tissue to perform quantitative reporter expression, as mesectoderm cells are easy to identify using an antibody to Sim. To do so, we generated a series of transgenic reporter lines containing varying numbers of CSL (*2x*, *4x*, *8x*, *or 12xCSL*_*17*_*-lacZ*) or SPS (*1x*, *2x*, *4x or 6xSPS*_*15*_*-lacZ*) sites and analyzed the activity of each in the mesectoderm of age-matched embryos using immunofluorescent imaging for both Sim and β-gal protein levels (see Materials and Methods). Analysis of reporters containing the same total number of Su(H) binding sites (1xSPS = 2xCSL) revealed that neither a single SPS site (*1xSPS*_*15*_*-lacZ*) nor two CSL sites (*2xCSL*_*17*_*-lacZ*) activated detectable reporter expression in the mesectoderm (**S8 Fig**). By contrast, Notch reporter activity in the mesectoderm was observed in embryos containing *lacZ* reporters with 4 or more CSL sites and 2 or more SPS sites (**Fig 5**). These data are consistent with previously published live imaging studies in the *Drosophila* mesectoderm [44] showing that the cooperative binding between NCM complexes does not confer SPS-containing enhancers with a significantly different response threshold to Notch activation in the mesectoderm from enhancers with the same number of independent CSL sites.

Next, we quantitatively assessed how the number and type of binding sites impact transcriptional output in the mesectoderm by analyzing *lacZ* reporter activity in two ways. First, we determined the percentage of mesectoderm cells (as defined by Sim positive staining) that expressed significant levels of β-gal relative to the background (defined as more than 3 standard deviations above the average background fluorescence, see Materials and Methods) (**Fig 5G**). Second, we measured the intensities of β-gal and Sim in each embryo as a function of binding site type (SPS vs CSL) and binding site number (**Fig 5H** and **5I**). As expected, Sim protein levels did not vary greatly between samples, although a small, but significant difference between the *2xSPS*_*15*_*-lacZ* and *4xCSL*_*17*_*-lacZ* samples was observed (**Fig 5I**). In contrast, comparative analysis of β-gal expression between these samples revealed the following: 1) Synthetic Notch reporters with SPS sites have a significantly higher likelihood of activating gene expression in each mesectoderm Sim positive cell than synthetic reporters with an equal number of independent CSL sites (**Fig 5G**). For example, the *4xSPS*_*15*_*-lacZ* reporter was activated in 78.0±6.3% (mean±sem) of mesectoderm cells in a typical embryo, whereas the *8xCSL*_*17*_*-lacZ* reporter was only activated in 53.6±6.7% of mesectoderm cells. Moreover, a similar significant difference was observed between the *6xSPS*_*15*_*-lacZ* and *12xCSL*_*17*_*-lacZ* embryos. 2) When comparing synthetic reporters with the same number of Su(H) binding sites (i.e. 8xCSL_17_ to 4xSPS_15_), the β-gal levels were significantly higher in the SPS reporter lines than those in the CSL reporter lines (**Fig 5H**). 3) There was a dramatic increase in both the percentage of β-gal-positive/Sim-positive cells (**Fig 5G**) and the levels of β-gal expression (**Fig 5H**) as the number of synthetic binding sites increased from 4xCSL_17_ to 8xCSL_17_ or from 2xSPS_15_ to 4xSPS_15_. However, both the levels of β-gal and the percentage of mesectoderm cells that activated Notch reporter activity were not significantly different between embryos with the *8xCSL*_*17*_*-lacZ* and *12xCSL*_*17*_*-lacZ* reporters or the *4xSPS*_*15*_*-lacZ* and *6xSPS*_*15*_*-lacZ* reporters (**Fig 5G** and **5H**), suggesting that Notch-mediated transcriptional activation plateaus above 8 CSL sites and 4 SPS sites. In sum, this analysis revealed that the synthetic SPS reporters are both more likely to be activated and express at higher levels than the synthetic CSL reporters with the same number of binding sites within the Notch-active mesectoderm cells.

### Activation of the SPS reporter gene is more resistant to increased levels of the Hairless co-repressor

**Fig 6:**
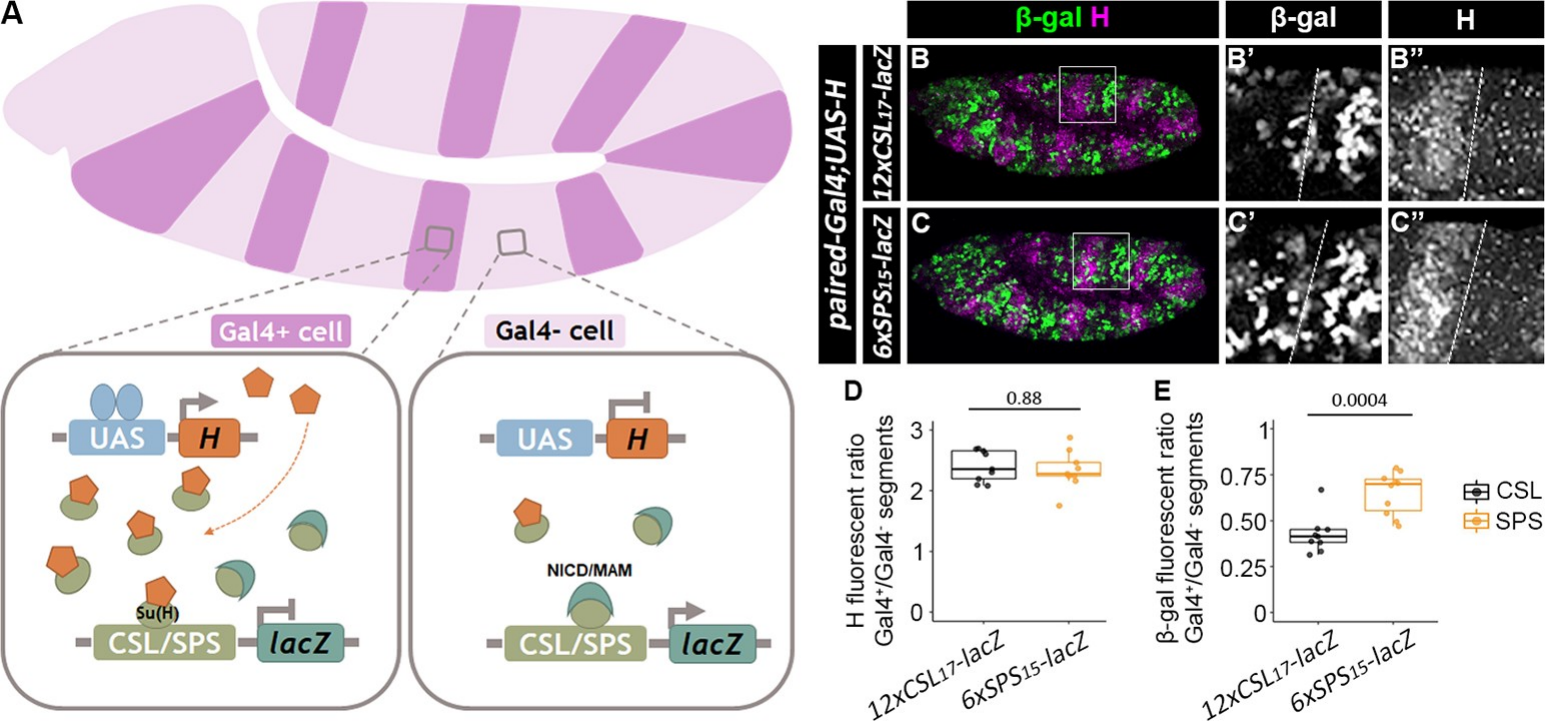
Notch reporters with cooperative binding sites show higher resistance to the Hairless co-repressor than reporters with monomer sites. **A**. Schematic of the over-expression of the Hairless protein using the *paired-Gal4>UAS* system. Note, that *paired-Gal4* is active in every-other parasegment and thereby allows the direct comparison of Gal4-positive (Gal4+) regions that express endogenous and exogenous Hairless with wild type (Gal4-) regions that only express endogenous Hairless in the same embryo. **B-C**. Lateral views of stage 11 *paired-Gal4>UAS-Hairless* embryos containing either the *12xCSL*_*17*_*-lacZ* (B) or *6xSPS*_*15*_*-lacZ* (C) reporter. Embryos were immunostained with β-gal (green) and Hairless (magenta) and close-up views of the individual channels in black and white for the highlighted regions are shown in B’-C’ (β-gal) and B”-C” (Hairless). Both *lacZ* transgenes were inserted into the ZH-51C locus. **D-E**. Quantification of ratios of Hairless (D) and β-gal (E) in parasegments with ectopic Hairless (*paired-Gal4*^*+*^) compared to control parasegments (*paired-Gal4*^*-*^). Each dot represents the mean measurement from an individual embryo containing either the *12xCSL*_*17*_*-lacZ* or *6xSPS*_*15*_*-lacZ* reporter. Sample sizes (n) were 9 for *12xCSL*_*17*_*-lacZ*, and 10 for *6xSPS*_*15*_*-lacZ*. Box plots show the median, interquartile range, and 1.5 times interquartile range. One-way ANOVA was used to test significance.

Because neither the NICD/Mam co-activators nor the H co-repressor has a DNA binding domain and they bind to Su(H) in a mutually exclusive manner, the activating complexes and repressing complexes likely compete for binding to enhancers to regulate gene expression [34,35]. To determine if the cooperativity between the NCM complex on the SPS results in altered sensitivity to the Hairless co-repressor, we overexpressed Hairless in every other parasegment of stage 11 embryos with *paired-Gal4;UAS-Hairless* (*PrdG4;UAS-H*) and analyzed age-matched embryos for either *12xCSL*_*17*_*-lacZ* or *6xSPS*_*15*_*-lacZ* reporter activity (see schematic in **Fig 6A**). Interestingly, while similar levels of Hairless overexpression were observed in both reporter lines compared to neighboring non-overexpressing parasegments (**Fig 6B, 6C** and **6D**, 2.41±0.08 fold with *12xCSL*_*17*_*-lacZ* and 2.43±0.13 fold with *6xSPS*_*15*_*-lacZ*, mean±sem), the *12xCSL*_*17*_*-lacZ* reporter was more effectively repressed by Hairless overexpression than the *6xSPS-lacZ* reporter (**Fig 6B, 6C** and **6E**, a 57.5±3.4% reduction in *12xCSL*_*17*_*-lacZ* activity versus a 35.0±3.7% reduction in *6xSPS*_*15*_*-lacZ* activity compared to the neighboring wild type parasegments, mean±sem). As a control, we found that expressing a *UAS-GFP* transgene with *PrdG4* did not significantly alter *12xCSL*_*17*_*-lacZ* and *6xSPS*_*15*_*-lacZ* reporter activity (**S9A, S9B** and **S9C Fig**). Moreover, to ensure that the distinct flanking sequences do not influence the differential responsiveness to Hairless, we again used *PrdG4* to ectopically express Hairless and analyzed the activity of the *6xSPS*_*17*_*-lacZ* reporter that has the same flanking sequences as the *12xCSL*_*17*_*-lacZ* reporter. Importantly, we found that ectopic Hairless expression again did not repress the *6xSPS*_*17*_*-lacZ* reporter (**S9D, S9E** and **S9F Fig**, a 9.1%±4.5% reduction in Hairless overexpressing segments compared to the neighboring wild type segments, mean±sem) nearly as well as the *12xCSL*_*17*_*-lacZ* reporter. (Note, the inverse experiment with the *12xCSL*_*15*_*-lacZ* reporter could not be performed as it is not expressed well in the embryo (**S9D Fig**)). These data are consistent with Su(H)/H complexes more effectively competing with the NCM co-activator for independent CSL sites than for cooperative SPS sites.

**Fig 7:**
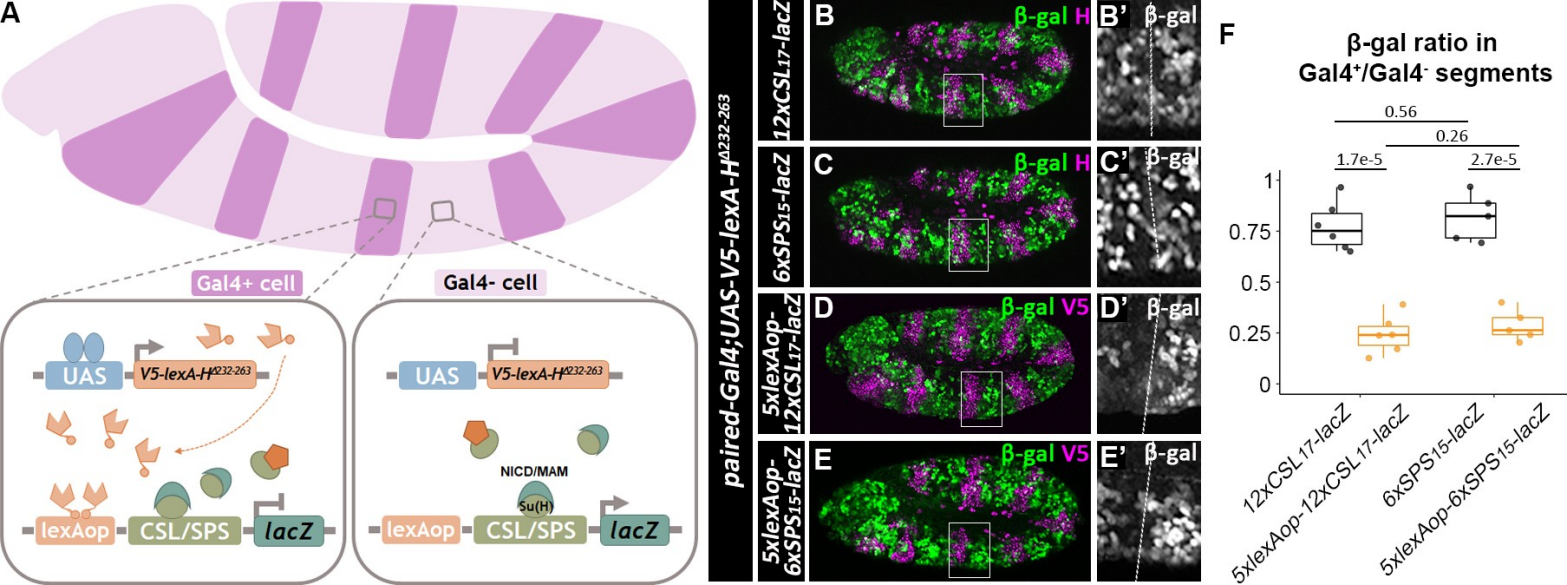
Hairless represses both cooperative and non-cooperative Notch-mediated transcriptional activation when targeted to DNA via a heterologous DNA binding domain. **A**. Schematic of the over-expression of a V5-lexA-HairlessΔ232–263 protein using the paired-Gal4-UAS system. Note, that the Hairless Δ232–263 deletion removes the protein domain that interacts with Su(H). Thus, this protein neither directly competes with NICD/Mam for binding to Su(H) nor does it get recruited to the CSL/SPS binding sites. Instead, the V5-LexA-HΔ232–263 protein is targeted to DNA via lexAop sequences that have been inserted into the CSL/SPS reporter vectors. **B-C**. Stage 11 embryos of *paired-Gal4>UAS-V5-lexA*^*DBD*^*-Hairless*^*Δ232–263*^ immunostained with β-gal (green) and Hairless (magenta) with either *12xCSL*_*17*_*-lacZ* or *6xSPS*_*15*_*-lacZ* reporter. B’-C’. Close-up views of β-gal intensity in black and white are shown in insets from B-C with the *paired-Gal4*-positive parasegment on the left and the *paired-Gal4*-negative parasegment on the right. **D-E**. Stage 11 embryos of *paired-Gal4>UAS-V5-lexA*^*DBD*^*-Hairless*^*Δ232–26*^ immunostained with β-gal (green) and V5 (magenta) with either *5xlexAop-12xCSL*_*17*_*-lacZ* or *5xlexAop-6xSPS*_*15*_*-lacZ*. D’-E’. Close-up views of β-gal intensity in black and white are shown in insets from D-E with the *paired-Gal4*-positive parasegment on the left and the *paired-Gal4*-negative parasegment on the right. **B-E**. Each *lacZ* transgene was inserted into the ZH-51C locus. **F.** Quantification of ratios of β-gal of paired-Gal4^+^ to paired-Gal4^-^ parasegments in flies with indicated genotypes. Each dot represents the average measurement from an individual embryo containing the indicated reporter. Sample sizes (n) were 6 for *12xCSL*_*17*_*-lacZ*, 5 for *6xSPS*_*15*_*-lacZ*, 7 for *5xlexAop-12xCSL*_*17*_*-lacZ*, and 5 for or *5xlexAop-6xSPS*_*15*_*-lacZ*. Box plots show the median, interquartile range, and 1.5 times interquartile range. One-way ANOVA used to test significance.

An alternative explanation for the increased resistance to Hairless of the *6xSPS*_*15*_*-lacZ* reporter is that when both Su(H)/H and NCM activation complexes are bound to neighboring sites on the same enhancer, the Su(H)/H complex may more efficiently antagonize the activation potential of the monomer NCM complex than that of the dimer NCM complex. To test this idea, we targeted the Hairless co-repressor to heterologous DNA binding sites using 5 copies of the LexA DNA binding site (5xLexAop) inserted adjacent to either 12xCSL_17_ or 6xSPS_15_ sites (**Fig 7A**). To do so, we generated a *UAS-V5-LexADBD-Hairless*^*Δ232–263*^ construct and overexpressed this fusion protein with *paired-Gal4* in reporter lines containing LexA operator binding sites (i.e. *5xlexAop-12xCSL*_*17*_*-lacZ* or *5xlexAop-6xSPS*_*15*_*-lacZ*). Deletion of the Hairless Δ232–263 amino acids removes the Su(H)-binding domain (Maier et al., 2011), and thus renders this protein incapable of being recruited to CSL or SPS sites. Hence, overexpressing the V5-LexADBD-H^Δ232–263^ protein had negligible impacts on the expression of the *12xCSL*_*17*_*-lacZ* and the *6xSPS*_*15*_*-lacZ* reporters lacking lexAop sites (**Fig 7B, 7C** and **7F**). In contrast, expressing the V5-LexADBD-H^Δ232–263^ protein strongly repressed the activity of both the *5xlexAop-12xCSL*_*17*_*-lacZ* and the *5xlexAop-6xSPS*_*15*_*-lacZ* reporters to a similar degree (**Fig 7D, 7E** and **7F**). As additional controls, expressing a V5-lexADBD protein that lacks the Hairless protein or expressing the V5-Hairless^Δ232–263^ protein that is not targeted to DNA failed to repress the *5xlexAop-12xCSL*_*17*_*-lacZ* and *5xlexAop-6xSPS*_*15*_*-lacZ* reporters (**S10 Fig**). Altogether, these data suggest that when Hairless is specifically targeted to DNA sites near where the NCM complex binds, it efficiently antagonizes NCM mediated activation regardless of site architecture. However, in wild type embryos where the Hairless/Su(H) complex and the NCM complex compete for binding sites, the cooperativity of the NCM complex for SPS sites makes these synthetic enhancers more resistant to Su(H)/H binding and repression.

## Discussion

In this study, we investigated how differences in DNA binding site architecture (CSL vs SPS) impact the DNA binding of the *Drosophila* Su(H) co-activator and co-repressor complexes *in vitro* and transcriptional output *in vivo*. Using a combination of *in vitro* DNA binding assays, synthetic biology, and *Drosophila* genetics, we made three key findings that reveal new insights into the differences between monomeric CSL sites and dimeric SPS sites in mediating Notch-dependent transcription. First, we found that unlike the Su(H)/NICD/Mam activating complex, the tested Su(H)/H repressor complex does not interact with SPSs in a cooperative manner and instead binds in a similar additive manner to both CSL and SPS probes. Second, while we failed to create a universal set of synthetic Notch reporters that are active in all known Notch dependent tissues due to the influence of flanking sequences, we did find that synthetic SPS enhancers are more consistently activated and activated to a higher level in the mesectoderm relative to the synthetic enhancer with equal numbers of monomeric CSL sites. Third, we found that the Hairless co-repressor can more readily repress Notch induced activation of the synthetic CSL enhancers than the synthetic SPS enhancers, whereas H could equally repress Notch mediated activation of both SPS and CSL sites when targeted to the enhancer via a heterologous DNA binding domain. Overall, these data support the model that, compared to enhancers with only CSL sites, Notch-regulated enhancers with cooperative SPSs will more likely be bound by the co-activator complex and thereby are more resistant to the potential negative impacts of CSL/co-repressor complexes. Below, we integrate these findings with other publications on Su(H) stability, Notch transcriptional dynamics, and endogenous Notch-regulated enhancers.

Recent studies in *Drosophila* have demonstrated that the Su(H) TF is unstable in the absence of either the Notch signal (NICD) or the Hairless co-repressor [59]. Moreover, biochemical assays demonstrated that Su(H), as well as the mammalian RBPJ CSL TF, uses distinct but overlapping domains to bind NICD and co-repressors and do so with similar affinities [35,37,60,61]. Together, these findings suggest that the NICD/Mam co-activator proteins and the Hairless co-repressor protein compete to bind Su(H) in a mutually exclusive manner, and that the vast majority of Su(H) in a cell is in either an activating or repressing complex. Hence, Notch-mediated transcriptional output is dependent upon which TF complex interacts with the binding sites found in Notch-regulated enhancers. In fact, live imaging studies have shown that stimulating Notch signaling results in both increased genomic accessibility and activity of Notch regulated enhancers within the *E(spl)* locus as well as increased binding of the Hairless co-repressor [42]. The counterintuitive finding that Hairless binding increases upon Notch stimulation supports the idea of assisted loading, which posits that an activator TF complex can increase the accessibility of DNA binding sites that can either bind additional co-activator or co-repressor complexes via the same DNA sites [42]. Importantly, our *in vitro* DNA binding data show that monomeric CSL sites bind similarly to both the Su(H)/NICD/Mam activating complex and the Su(H)/H repressing complex with similar affinities and kinetics (**Figs 1 and 2** and **S1**). In contrast, the paired sites found in SPS enhancers cooperatively bind the Su(H)/NICD/Mam complex, but not the Su(H)/H co-repressor complex, and our quantitative biochemical studies show that the effective Kd for binding a second co-activator complex to the SPS site is ~17 times smaller than the effective Kd of binding a second Su(H)/H co-repressor complex or a second Su(H) TF alone to the SPS site. However, since a truncated Hairless protein was used in our EMSAs, we cannot rule out the possibility that regions outside of the tested construct contribute to cooperativity. But importantly, the DNA binding data are congruent with our reporter data showing that SPS enhancers are more resistant to Hairless overexpression as compared to CSL enhancers integrated into the same chromosomal locus.

Previous studies on Notch-dependent transcription in the mesectoderm used the MS2-MCP:GFP system to characterize the transcriptional dynamics of two enhancers: the *E(spl)m5/m8* mesectoderm enhancer (MSE) that has an SPS site as well as several potential monomeric CSL sites, and the *sim* MSE enhancer that lacks SPSs but contains monomeric CSL sites [44]. Interestingly, the *E(spl)m5/m8* MSE and *sim* MSE enhancers showed very similar transcriptional dynamics and highly correlated transcriptional activity, suggesting that SPS and CSL sites mediate similar transcriptional responses within the mesectoderm. However, when different doses of ectopic NICD were provided in neighboring cells, the *E(spl)m5/m8* MSE enhancer drove expression significantly earlier than the *sim* MSE, consistent with the notion that the *E(spl)m5/m8* MSE enhancer displays a lower detection threshold for NICD to activate transcription. Intriguingly, this difference in enhancer activity was likely due to additional TF inputs and not due to the SPS site, as neither converting the SPS into 2 CSL sites within the *E(spl)m5/m8* MSE nor adding an SPS to the *sim* MSE changed the timing of their activity. In contrast, both the SPS-containing wild type *E(spl)m5/m8* MSE enhancer and the engineered SPS-containing *sim* MSE enhancer were found to activate higher levels of gene expression due to increased transcriptional burst size.

Our findings using synthetic enhancers are largely in agreement with the results obtained using live imaging in *Drosophila* embryos [44]. First, we found that synthetic SPS and CSL enhancers both required the same number of Su(H) binding sites (*2xSPS* vs. *4xCSL*) to activate reporter expression within the mesectoderm, whereas the *1xSPS-lacZ* and *2xCSL-lacZ* reporters both failed to activate gene expression in the mesectoderm. This finding is in line with SPS and CSL sites having similar NICD detection thresholds. Second, we found that SPS enhancers activated transcription more consistently and at a higher level than CSL enhancers with the same total number of binding sites. Third, we investigated if the SPS-mediated cooperativity grants the *Drosophila* co-activators any advantages over the co-repressors and found that only the NCM complex, but not the Su(H)/H co-repressor complex, cooperatively binds SPSs. Consistent with the idea that cooperative binding to SPSs may lead to increased resistance to changes in co-repressor levels, our reporter assays showed that when Hairless was overexpressed at a moderate level (~2-fold overexpression) and had to compete with co-activators for Su(H) binding, the SPS reporter was more resistant to Hairless than the CSL reporter. However, when we targeted Hairless to DNA via an independent non-competitive mechanism, the Hairless co-repressor was equally competent to antagonize the activation effects elicited by the NCM complex on either the synthetic CSL or SPS reporters. Thus, the co-activator and co-repressor complexes compete for binding sites, and the cooperativity of the NCM to SPSs results in a competitive advantage for the activation complex over the repression complex.

Integrating our findings using the synthetic SPS and CSL enhancers with the studies on transcription dynamics of endogenous enhancers supports the following model: The cooperative binding of NCM activating complexes on SPSs results in enhanced stability of the NCM complex (i.e. a longer half-life as measured by our temporal EMSA competition assays, see **Fig 2**) relative to independent CSL sites, which results in larger transcriptional burst sizes and enhanced levels of gene expression via SPS-containing enhancers. In addition, cooperative NCM binding to SPS enhancers renders these binding sites less sensitive to the repressive impacts of the Su(H)/Hairless co-repressor complex. Importantly, each of these properties (i.e. cooperative NCM binding and preferential co-activator binding to DNA over co-repressor binding) would result in more consistent and higher transcription levels of target genes. However, there are several factors to consider regarding how these differences in synthetic SPS versus CSL enhancer activity can be translated to endogenous Notch regulated enhancers. First, most endogenous enhancers with an SPS also contain one or more independent CSL sites that are more highly sensitive to the Hairless co-repressor. Hence, the transcriptional dynamics and ultimate output of endogenous Notch enhancers are likely to be influenced by the combined number and accessibility of the SPS versus CSL binding sites. Second, we only tested two spacer lengths between the Su(H) sites (15bp and 17bp) in the head-to-head (SPS) and head-to-tail (CSL) orientations, and it is certainly possible that other distinct Su(H) binding site orientation/spacing may influence the transcriptional outcome. For example, Ozdemir et al found that a synthetic enhancer containing two Su(H) sites organized head-to-tail (CSL) and spaced 5bp apart were sufficient to repress transcription in the early *Drosophila* embryo without mediating obvious gene activation in Notch-active mesectoderm cells [62]. Since it is currently unclear if this orientation/spacing is generally more strongly associated with repression over activation, future studies will be required to determine how other spacing/orientation parameters between Su(H) sites can influence transcriptional outcomes. Third, even though we designed our synthetic SPS and CSL enhancers to specifically limit other TF inputs, we found that the flanking sequences can have a profound impact on Notch transcriptional output in complex tissue-specific ways that are not well understood. For example, while our synthetic SPS_15_ and CSL_15_ enhancers were expressed in many expected Notch-dependent patterns in larval imaginal disc tissues, the SPS_17_ and CSL_17_ enhancers failed to convey expression in the larval imaginal discs. However, we found that in the embryo and pupal eye discs, the SPS_17_ and CSL_17_ enhancers activated more consistent Notch expression patterns than the SPS_15_ and CSL_15_ enhancers. Moreover, we found that simply “flipping” one of the Su(H) sites to convert a synthetic SPS into a CSL or a synthetic CSL into an SPS could create new predicted TFBS motifs (**S3 Fig**) and could result in altered ectopic expression patterns (**Figs 3** and **4**). These data highlight the challenge in using synthetic biology to create a universal Notch reporter that has either SPS or CSL sites that are not strongly influenced by the adjacent sequences. Importantly, this finding is consistent with the fact that even widely activated endogenous Notch target genes such as the *E(spl)* genes are not activated in all Notch-dependent cell types [23,55]. Thus, these findings highlight the difficulty, if not impossibility, to design universal Notch activated enhancers that are active in all Notch-dependent cell types.

## Materials and methods

### Protein purification and electrophoretic mobility shift assays (EMSAs)

*Drosophila* proteins used in EMSAs include Su(H) (aa 98–523), Hairless (aa 232–358), NICD (aa 1763–2142) and Mastermind (aa 87–307). Recombinant proteins of each were expressed in *E*. *coli* and purified using affinity (Ni-NTA or Glutathione) ion exchange and size exclusion chromatography as previously described [60]. The purity of proteins was determined by SDS-PAGE with Coomassie blue staining and protein concentration was measured by UV280 absorbance. All EMSAs were performed essentially as previously described [63,64]. In brief, fluorescent labeled probes were mixed with purified proteins and incubated at room temperature for 20 minutes before loading except for the competition assays with unlabeled probes. For the temporal competition assays, the indicated proteins and fluorescent labeled probes were mixed and incubated at room temperature for 10 minutes. Samples were then transferred to a 4°C water bath for 10 minutes. 10x of unlabeled 2xCSL_17_ competitor DNA was subsequently added and samples were loaded into the acrylamide gels at the indicated time points. The protein concentration used for each experiment is listed in each Figure legend. Probe sequences are listed in S1 Table. Acrylamide gels were run at 150V for 2 hours and then imaged using the LICOR Odyssey CLx scanner.

### EMSA quantification

The raw data for the mathematical analysis was either extracted from gray scale images of the EMSA gels (for the cooperativity factor) or using Image Studio software (for the dissociation rates). For calculating the cooperativity factor, the entire process was performed with custom MATLAB code. We utilized a local minima algorithm to extract the inter-lane intensity values. Inter-lane values were used to fit the appropriate background value to a specific location in the image. Band values were extracted by calculating the background subtracted intensity sum over rectangular boxes, which were optimized for maximal signal to background.

We then fitted the extracted band intensities to an equilibrium model for binding two sites in a cooperative manner, in a similar way as described in Kobia et al. [30]. In short, the binding probabilities were calculated using standard binding kinetics (Michaelis-Menten). The probabilities that the probe is bound by 0, 1 or 2 complexes are given by:

$$P_{0}=\frac{1}{1+2\alpha +C\alpha^{2}},P_{1}=\frac{2\alpha }{1+2\alpha +C\alpha^{2}},P_{2}=\frac{C\alpha^{2}}{1+2\alpha +C\alpha^{2}}$$

where $\alpha =\frac{\left[TF\right]}{K_{d}}$ is the statistical weight associated with binding of a TF complex to a CSL or SPS site. *K*_*d*_ is the dissociation constant to a single site. The cooperativity factor, *C*, describes the factor by which the binding affinity for the second site changes relative to binding affinity of the first site, namely $K_{d2}=\frac{1}{C}K_{d}$. The case of *C* = 1 corresponds to a non-cooperative binding. *C*>1 corresponds to positive cooperativity (2^nd^ binding is enhanced). *C*<1 Corresponds to negative cooperativity (2^nd^ binding is suppressed). We observed that even at high concentrations of Su(H) the 1-site state is never depleted (e.g. see NCM on SPS), and the signal of the 0-site state never decays to zero. We therefore assumed that there is a probability, *f*, that a site will become unavailable for binding. Under this assumption there is a fraction *f*^2^ of the probes, that will have no functioning sites (i.e. that both sites are unavailable), and a fraction 2*f* of the probes that have only 1 functioning site (one of the two sites is unavailable). In this case the probability to find the probe is modified to:

$$2‐sites:\;P_{2}=(1-2f-f^{2})\frac{C\alpha^{2}}{1+2\alpha +C\alpha^{2}}.$$

$$1‐site:\;P_{1}=(1-2f-f^{2})\frac{2\alpha }{1+2\alpha +C\alpha^{2}}+2f\frac{\alpha }{1+\alpha }.$$

$$0‐sites:\;P_{0}=(1-2f-f^{2})\frac{1}{1+2\alpha +C\alpha^{2}}+2f\frac{1}{1+\alpha }+f^{2}.$$

We then fit the normalized band intensities using least mean squares to the sum of these three expressions. The fitting parameters are *K*_*d*_, *C*, and *f*. The parameters are extracted for each experiment separately.

The confidence interval on the fitting parameters was calculated using a bootstrap method where 5000 random data sets with the same mean and standard deviation as those observed experimentally were generated. The fitting procedure was then applied to all bootstrapped data to obtain the distribution of fitting parameters. The confidence intervals were determined by calculating the 95-percentile range for each parameter.

For calculating the half-lives from the temporal competition EMSA measurements, we extracted the 2NCM and 2Su(H)/H bands from the 4 replicates. The bands from each replicate were normalized to the band at t = 0. The data from all 4 replicates was fitted to a decaying exponent functions of the form $f=(1-c_{1})∙e^{-t∙c_{2}}+c_{1}$, where the parameters *c*_1_ and *c*_2_ correspond to the constant background level and the decay rate, respectively. Half-lives were calculated from *c*_2_ using the formula $t_{1/2}=\frac{ln2}{c_{2}}$. Fitted parameter values and corresponding 95% confidence intervals were extracted using a custom Matlab code (https://github.com/Eafergan/EMSA-Kuang-et-al.git).

### Generation of transgenic flies

12xCSL_17_ and 6xSPS_15_ synthetic enhancers were designed and synthesized by anchoring two consensus Su(H) sites (CGTGGGAA) 15-17bps apart in either a head-to-tail (CSL) or head-to-head (SPS) manner. The flanking sequences were randomly generated 1000 times each and each probe was searched for *Drosophila* TFBS motifs using CIS-BP. The 1xSPS_15_ and 2xCSL_17_ sequences with the fewest additional TFBS motifs were selected for further analysis. The 12xCSL_15_ was created by inverting one of the Su(H) sites (i.e. reverse complementation) in the 6xSPS_15_ sequence. The 6xSPS_17_ sequence was created by inverting one of the Su(H) sites in the 12xCSL_17_ sequence. The *12xCSL*_*17*_, *12xCSL*_*17*_*mut*, *12xCSL*_*15*_, *6xSPS*_*15*_, *6xSPS*_*15*_*mut*, and *6xSPS*_*17*_ sequences were all synthesized by Genscript and cloned into the *placZ-attB* vector [47]. The 2xCSL_17_, 4xCSL_17_, 8xCSL_17_, 1xSPS_15_, 2xSPS_15_, and 4xSPS_15_ sequences were synthesized as oligonucleotides containing appropriate restriction enzyme site overhanging sequences to aid cloning into the *placZ-attB* vector. The 5xlexAop sequence was synthesized by Genscript with flanking HindIII and EcoR1 restriction enzyme sites to aide cloning into the following vectors: *placZ-attB*; *12xCSL*_*17*_*-lacZ* or *6xSPS*_*15*_*-lacZ*. The coding sequences for the LexA-DBD and Hairless^Δ232–263^ sequences were synthesized by Genscript with appropriate flanking restriction enzyme sites for cloning into a modified pUAST vector that contained an N-terminal V5-epitope tag. These synthesized DNAs were used to generate the pUAST-V5-lexADBD, pUAST-V5-Hairless^Δ232–263^, and pUAST-V5-LexADBD-Hairless^Δ232–263^ vectors. All sequences were confirmed by Sanger sequencing, purified using Qiagen Midi-prep Kit and sent for *Drosophila* injection to Rainbow Transgenic, Inc. Transgenic *Drosophila* lines were established by integration into the *Drosophila* genome using phiC31 recombinase integrase and landing sites located at either 51C or 86Fb as indicated [47]. All newly derived sequences and restriction sites used for cloning are listed in S2 Table.

### Fly husbandry

The following alleles were obtained from the Bloomington *Drosophila* Stock Center: *paired-Gal4* (#1947), *UAS-NICD* (#52008), and *UAS-Hairless* (#15672). Flies were maintained at 25°C and under standard conditions.

### Generation of single-minded (sim) antibody

Guinea pig anti-Sim serum was generated as previously described [65]. Briefly, a Sim cDNA was gifted from Dr. Stephen Crews (University of North Carolina). The cDNA sequence corresponding to sim-PD (aa 361–672) was PCR amplified and cloned in-frame with a 6xHis-Tag into a modified pET-14b plasmid (Novagen). The expression plasmid was transformed into BL21 competent *E*. *coli* and the expression of the fusion protein was induced by IPTG. The His-tag-Sim protein was extracted in 8M urea lysis buffer, purified by Ni-NTA affinity chromatography, confirmed by Coomassie blue staining, and injected into guinea pigs to generate anti-Sim serum (Cocalico Biologicals, Inc).

### Immunostaining and quantitative analysis

Embryos were harvested, fixed, and immunostained following previous published protocols [66]. In brief, for analysis of the early *Drosophila* embryos (i.e to image mesectoderm cells), homozygous flies of the indicated genotypes were allowed to lay eggs on apple-agar plates for two hours. The embryo-containing plates were then removed from the cages and allowed to develop for an additional two hours at 25°C. These 2–4 hour-old *Drosophila* embryos were collected, fixed for 20 min in 4% paraformaldehyde/PBS with vigorous shaking (180 RPMs), and immunostained using anti-β-gal (chicken 1:1000, Abcam ab9361) and anti-Sim (guinea pig 1:500, this study) serum and appropriate secondary antibodies conjugated to fluorescent dyes (Jackson Labs). Embryos that were undergoing invagination were selected based on the separation of the two sim-positive stripes and imaged under identical settings using a Nikon A1R inverted confocal microscope (20x objective) or a ZEISS Apotome microscopy. The mesectoderm cells were defined as Sim-positive and selected for quantitative expression analysis. To do so, pixel intensity of both Sim and β-gal was subsequently determined with background correction using Imaris software. β-gal positive cells were defined as cells with pixel intensity at least three-fold higher than the standard deviation of the background measurements. One-way ANOVA with proper post-hoc tests was used to determine statistical significance.

For the *paired-Gal4* experiments, 0–16 hour-old embryos were collected, fixed as above, and immunostained with the indicated antibodies. Antibodies used in these experiments were Hairless (guinea pig 1:500, Annett and Dieter), β-gal (chicken 1:1000, Abcam ab9361), V5 (mouse 1:500, Invitrogen R960-25) and GFP (rabbit 1:500, Thermo Fisher A-11122) as indicated. Stage 11–12 *Drosophila* embryos were imaged under identical settings in each experiment by either a ZEISS Apotome or Nikon A1R inverted confocal microscope. Fluorescent intensity was quantified using Fiji software as previously described [67,68]. Briefly, the z-stack images were sum-projected and the Gal4^+^ and Gal4^-^ regions in embryos were manually determined. Gal4-positive segments were masked by quadrilateral in Fiji software based on the expression of either Hairless or V5, and Gal4-negative segments were defined as the regions in between the Gal4-positive segments. For each embryo, five segments starting from the first thoracic segment were quantified. Mean fluorescent intensities of each segment were collected using Fiji software, and for each embryo, data from three Gal4-positive segments and two Gal4-negative segments were averaged, respectively. After background subtraction, the ratio of β-gal and Hairless in Gal4-positive over Gal4-negative segments was calculated using the averaged data from each embryo. One-way ANOVA with proper post-hoc tests was used to determine statistical significance.

The larval wing discs [69], pupal eye discs [70], and adult posterior midguts [71] were each dissected and fixed as previously described in the respective references. All of the samples were stained as previously described [69]. Antibodies used in this study include β-gal (chicken 1:1000, Abcam ab9361) and sim (guinea pig 1:500, this study), cut (mouse 1:50, DSHB 2B10), Pros (mouse 1:100, DSHB MR1A), Hairless (guinea pig 1:500, Annett and Dieter) and V5 (mouse 1:500, Invitrogen R960-25).

## Supporting information

S1 Fig EMSA quantification and modeling of DNA binding states. **A-B**. Individual channels of the same EMSA data shown in **Fig 1D**, **C-H**. Quantification of the amount of probe that was not bound (unoccupied, red line), bound by a single complex (green line), and bound by two complexes (fully occupied, blue lines). The data for the 2xCSL_17_ probe is shown at left, whereas the data for the 1xSPS_15_ probe is shown at right. The concentration of Su(H) used is shown along the X-axis. **C-D**, Su(H) was added to each reaction in the absence of either the co-activator or co-repressor proteins. **E-F**, Su(H) was added to each reaction with an excess of the Hairless co-repressor**. G-H**, Su(H) was added to each reaction with an excess of NICD and Mam (NCM). Data points were extracted from EMSAs and represented as asterisks. Simulated data from the model are represented in lines. *C*, cooperativity factor. *K*_*d*_, equilibrium dissociation constant. f, fraction of sites unavailable for binding. Data are from four EMSA gels.
(TIF)

S2 Fig Specificity of 1xSPS_15_, 2xCSL_17_ and 2xCSL_17_mut probes as unlabeled competitors for Su(H). EMSA data reveals that the addition of the unlabeled 1xSPS_15_ and 2xCSL_17_ probes, but not the 2xCSL_17_mut probe results in decreased Su(H) binding to the labeled 2xCSL_17_ probe. 3.5nM labeled probe and 40nM Su(H) was used in indicated lanes. Three concentrations of each unlabeled competitor probe were tested with increases from 8.75nM to 140nM in four-fold steps.
(TIF)

S3 Fig cisBP analysis of 2xCSL_17_, 1xSPS_15_, 2xCSL15, and 1xSPS17 sequences for additional transcription factor binding site motifs. **A-B**. Transcription factor binding site prediction analysis of the 2xCSL_17_ and 1xSPS_15_ sequences for all known *Drosophila* TFs listed in the cis-BP database using a log-odds position weight matrix (PWM) score of 9 or higher. Note, the core 8bp sequence of the two Su(H) sites are highlighted in green and the other potential TF motifs are indicated by the black bars. **C.** SNP analysis between 2xCSL_17_ (A1 sequence, top) and 1xSPS_17_ (A2 sequence, top) and between 1xSPS_15_ (A1 sequence, bottom) and 2xCSL_15_ (A2 sequence, bottom) predicts acquired (A2 only) and/or lost (A1 only) transcription factor binding sites after inverting one of the Su(H) binding sites in 2xCSL_17_ or 1xSPS_15_.
(TIF)

S4 Fig CSL and SPS probes with different flanking sequences show similar behaviors in EMSAs. **A**. Sequences of the original 2xCSL_17_ and 1xSPS_15_ probes as well as the inverted 1xSPS_17_ and 2xCSL_15_ sequences with the orientation of each Su(H) site in each probe highlighted by an arrow. **B**. EMSAs reveal binding of purified Su(H) to the indicated probes. Su(H) concentration increases from 2.5nM to 160nM in 4-fold steps. **C**. EMSAs reveal binding of indicated purified proteins on 2xCSL_15_ and 1xSPS_17_ probes. Su(H) concentration increases from 2.5 to 160 nM in 4-fold steps and 2μM of either Hairless or NICD/MAM was used in the indicated lanes.
(TIF)

S5 Fig Mutating the Su(H) binding sites abolishes transcriptional activity from the CSL and SPS reporters in *Drosophila* tissues. **A-B**. Stage 5 *Drosophila* embryos containing either the *12xCSL*_*17*_*-lacZ* or the *12xCSLmut*_*17*_*-lacZ* reporter were immunostained and imaged under identical conditions for β-gal (green, black and white in A’ and B’) and Sim (magenta). Note, the mesectoderm expression activity of the *12xCSL*_*17*_*-lacZ* reporter is lost when the CSL binding sites were mutated. **C-D**. Larval wing discs containing either the *6xSPS*_*15*_*-lacZ* or the *6xSPSmut*_*15*_*-lacZ* reporter were immunostained and imaged under identical conditions for β-gal (green, black and white in C’ and D’) and Cut (magenta). Note, the wing margin cell expression activity of the *6xSPS*_*15*_*-lacZ* reporter is lost when each SPS binding site was mutated. Each *lacZ* transgene was inserted into the ZH-86Fb locus.
(TIF)

S6 Fig *12xCSL_17_-lacZ* and *6xSPS_15_-lacZ* transgenic reporters behave similarly in two different loci. **A**-**D**. Stage 15 *Drosophila* embryos homozygous for either the *12xCSL*_***17***_*-lacZ* (A-B) or *6xSPS*_***15***_*-lacZ* (C-D) at the indicated genomic loci (51C or 86Fb) were immunostained with β-gal. Note, the similar expression patterns by both transgenes in each chromosomal location. **E**-**H**. Third instar larval wing imaginal discs homozygous for the indicated reporters were immunostained with β-gal. Note, only the *6xSPS*_***15***_*-lacZ* reporter is active in the wing margin cells, whereas the *12xCSL*_***17***_*-lacZ* fails to activate significant gene expression when inserted into either the 51C or 86Fb locus.
(TIF)

S7 Fig Synthetic CSL and SPS reporters with distinct flanking sequences significantly differ in expression activity in larval imaginal discs. **A.** Schematics of the CSL and SPS reporter constructs with the orientation of each Su(H) binding site highlighted by an arrow. **B-I**. β-gal immunostaining of third instar larval imaginal discs reveals that the SPS and CSL reporters containing a 15bp spacer (C,E,G,I), but not the SPS and CSL reporters containing a 17bp spacer (B,D,F,H), are active in the expected pattern in larval leg discs (B-E) and larval eye-antenna discs (F-I). The *12xCSL*_*17*_*-lacZ* and *6xSPS*_*15*_*-lacZ* transgenes were inserted into the ZH-86Fb locus, and the *12xCSL*_*15*_*-lacZ* and *6xSPS*_*17*_*-lacZ* transgenes were inserted into the ZH-51C locus.
(TIF)

S8 Fig 2xCSL_17_ and 1xSPS_15_ reporters fail to mediate Notch activation in mesectoderm cells. **A-B**. Stage 5 *Drosophila* embryos containing either the *2xCSL*_**17**_*-lacZ* (A) or *1xSPS*_**15**_*-lacZ* (B) reporter were immunostained and imaged under identical conditions for β-gal (green, black and white in A’ and B’) and Sim (magenta). Note, neither reporter activates in the mesectoderm. Both *lacZ* transgenes were inserted into the ZH-86Fb locus.
(TIF)

S9 Fig Analysis of synthetic SPS and CSL reporter activity under conditions of either GFP or Hairless overexpression. **A-B.** Lateral views of stage 11 *paired-Gal4>UAS-GFP* embryos containing either the *12xCSL*_*17*_*-lacZ* (A) or *6xSPS*_*15*_*-lacZ* (B) reporter inserted into the ZH-51C locus. Embryos were immunostained with β-gal (green) and GFP (magenta), and close-up views of the individual channels in black and white for the highlighted regions are shown in A’-B’ (β-gal) and A”-B” (GFP). **C**. Quantification of the ratio of β-gal and GFP in parasegments with ectopic GFP (*paired-Gal4*^*+*^) compared to control parasegments (*paired-Gal4*^*-*^). Each dot represents the mean measurement from an individual embryo containing either the *12xCSL*_*17*_*-lacZ* or *6xSPS*_*15*_*-lacZ* reporter. Sample size (n) is 20 for *12xCSL*_*17*_*-lacZ* and 16 for *6xSPS*_*15*_*-lacZ*. Box plots show the median, interquartile range, and 1.5 times interquartile range. One-way ANOVA was used to test significance. These data show that ectopic expression of GFP by paired-Gal4 does not dramatically impact either *12xCSL*_*17*_*-lacZ* or *6xSPS*_*15*_*-lacZ* activity. **D-E**. Lateral view of stage 11 *paired-Gal4>UAS-Hairless* embryos containing either the *12xCSL*_*15*_*-lacZ* (D) or *6xSPS*_*17*_*-lacZ* (E) reporter. Embryos were immunostained with β-gal (green) and Hairless (magenta), and close-up views of the individual channels in black and white for the highlighted regions are shown in D’-E’ (β-gal) and D”-E” (Hairless). Note, because the *12xCSL*_*15*_*-lacZ* reporter is not active in the PrdG4 parasegments in the *Drosophila* embryo, we were unable to assess the impact of Hairless overexpression on this reporter. **F**. Quantification of ratios of β-gal and Hairless in parasegments with ectopic Hairless (*paired-Gal4*^*+*^) compared to control parasegments (*paired-Gal4*^*-*^). Each dot represents the mean measurement from an individual embryo containing *6xSPS*_*17*_*-lacZ* reporter. Sample size (n) is 10. Box plots show the median, interquartile range, and 1.5 times interquartile range. Note, unlike the *12xCSL*_*17*_*-lacZ* reporter expression that was strongly decreased by Hairless overexpression (see **Fig 6**), the *6xSPS*_*17*_*-lacZ* reporter with the same flanking sequences was not strongly impacted by ectopic Hairless. Each *lacZ* transgene was inserted into the ZH-51C locus.
(TIF)

S10 Fig lexA-H^Δ232–263^ fusion protein, but not lexA or H^Δ232–263^ alone, represses the *lacZ* reporters driven by lexA operator and CSL/SPS binding sites. **A-C**. Stage 11 embryos of *paired-Gal4;5xlexAop-12xCSL*_*17*_*-lacZ* with either *UAS-V5-Hairless*^*Δ232-263*^(A), *UAS-V5-lexA* (B) or *UAS-V5-lexA-Hairless*^*Δ232-263*^(C) immunostained for β-gal (green) and the V5 epitope (magenta). A’-C’. Close-up views of β-gal intensity in black and white are shown in insets from A-C with the *paired-Gal4*-positive parasegment on the left and the *paired-Gal4*-negative parasegment on the right. **D**. Quantification of ratios of β-gal of *paired-Gal4*-positive over *paired-Gal4*-negative parasegments in *paired-Gal4;5xlexAop-12xCSL*_*17*_*-lacZ* flies with indicated UAS construct. Each dot represents the average measurement from an individual embryo. Sample sizes (n) are 37 for *UAS-V5-Hairless*^*Δ232–263*^, 30 for *UAS-V5-lexA*, and 17 for *UAS-V5-lexA-Hairless*^*Δ232–263*^. Box plots show the median, interquartile range, and 1.5 times interquartile range. One-way ANOVA with post-hoc Tukey HSD was used to test significance. **E-G**. Stage 11 embryos of *paired-Gal4;5xlexAop-6xSPS*_*15*_*-lacZ* with either *UAS-V5-Hairless*^*Δ232-263*^(E), *UAS-V5-lexA* (F) or *UAS-V5-lexA-Hairless*^*Δ232-263*^(G) immunostained for β-gal (green) and the V5 epitope (magenta). E’-G’. Close-up views of β-gal intensity in black and white are shown in insets from E-G. **H**. Quantification of ratios of β-gal of *paired-Gal4*-positive over *paired-Gal4*-negative parasegments in *paired-Gal4;5xlexAop-6xSPS*_*15*_*-lacZ* flies with indicated UAS construct. Each dot represents the average measurement from an individual embryo. Sample sizes (n) are 13 for *UAS-V5-Hairless*^*Δ232–263*^, 14 for *UAS-V5-lexA*, and 13 for *UAS-V5-lexA-Hairless*^*Δ232–263*^. Box plots show the median, interquartile range, and 1.5 times interquartile range. One-way ANOVA with post-hoc Dunnett’s T3 was used to test significance. **A-G**. All the *lacZ* transgenes were inserted into ZH-51C locus.
(TIF)

S1 Data Spreadsheet containing all the raw data used to generate the graphs within the manuscript. (XLSX)

S1 Table List of oligonucleotide sequences used to generate the labeled probes used in the electrophoretic mobility shift assays. (DOCX)

S2 Table List of DNA sequences used to generate all the synthetic reporter and expression transgenic constructs. (DOCX)
