# Supplementary figures and images for: Enhancers with cooperative Notch binding sites are more resistant to regulation by the Hairless co-repressor
Source: PLoS Genet. 2021 Sep 24;17(9):e1009039. doi: 10.1371/journal.pgen.1009039 (PMC8494340; doi:10.1371/journal.pgen.1009039)

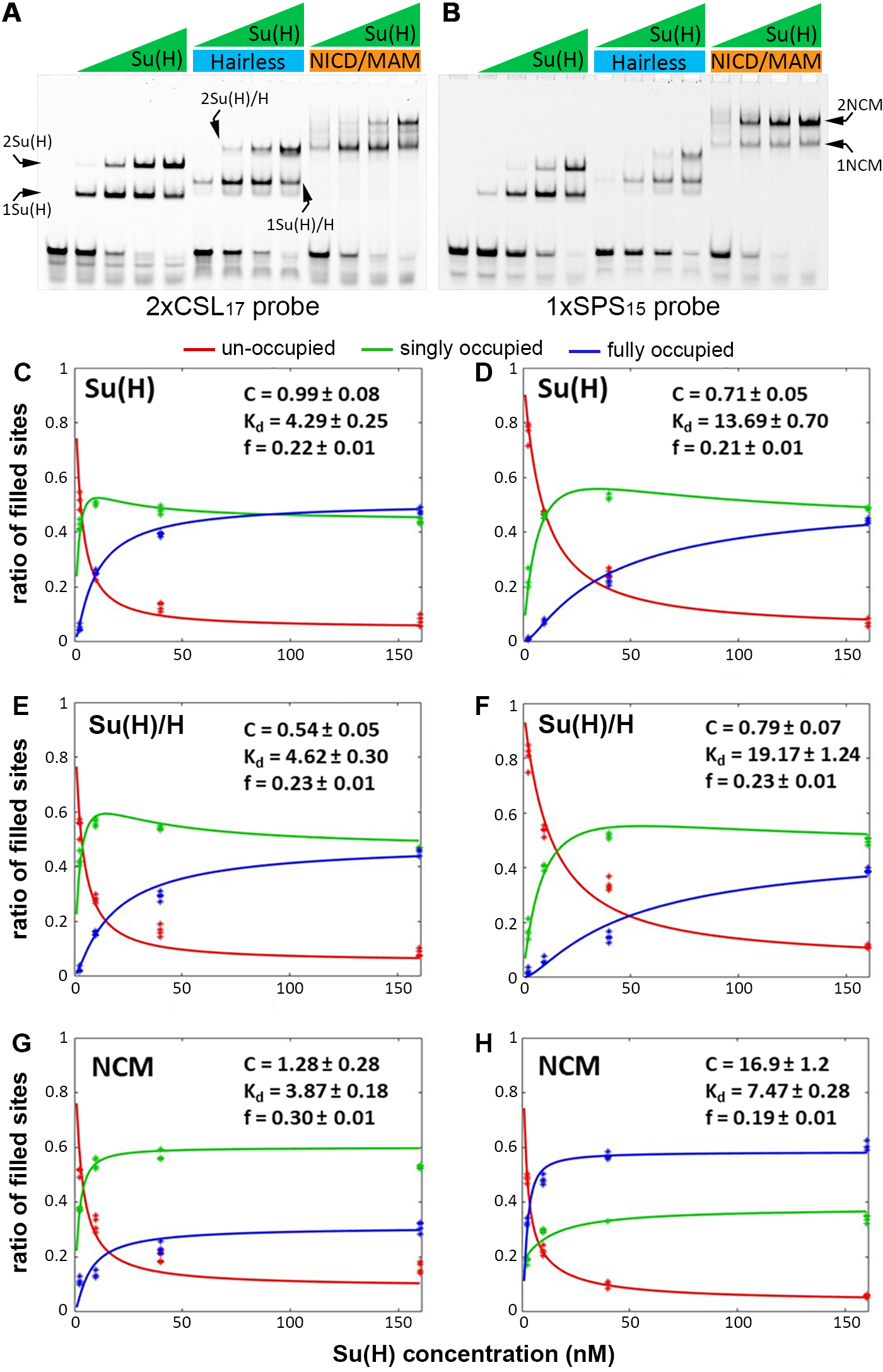

Supplement: S1 Fig — A-B. Individual channels of the same EMSA data shown in Fig 1D, C-H. Quantification of the amount of probe that was not bound (unoccupied, red line), bound by a single complex (green line), and bound by two complexes (fully occupied, blue lines). The data for the 2xCSL17 probe is shown at left, whereas the data for the 1xSPS15 probe is shown at right. The concentration of Su(H) used is shown along the X-axis. C-D, Su(H) was added to each reaction in the absence of either the co-activator or co-repressor proteins. E-F, Su(H) was added to each reaction with an excess of the Hairless co-repressor. G-H, Su(H) was added to each reaction with an excess of NICD and Mam (NCM). Data points were extracted from EMSAs and represented as asterisks. Simulated data from the model are represented in lines. C, cooperativity factor. Kd, equilibrium dissociation constant. f, fraction of sites unavailable for binding. Data are from four EMSA gels. (TIF) [file pgen.1009039.s001.tif]

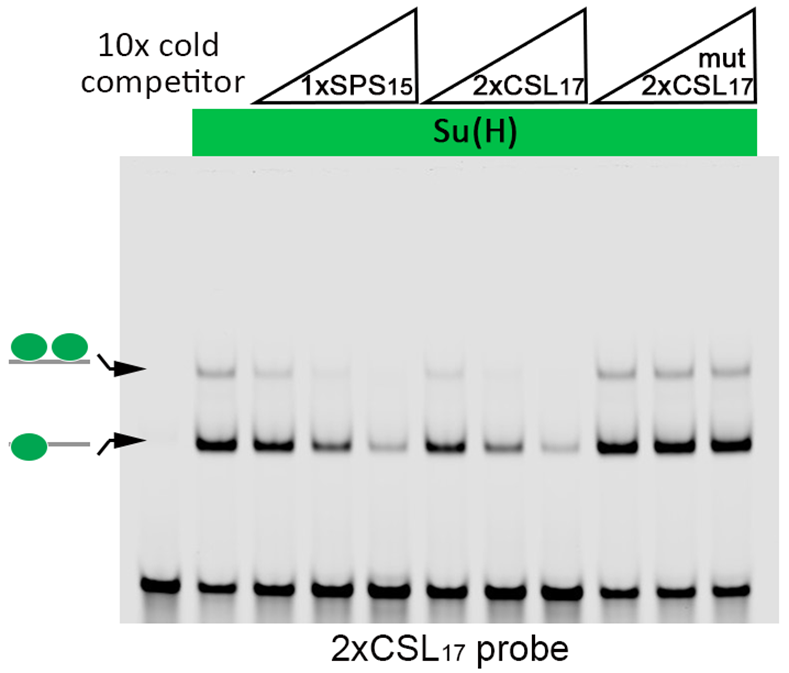

Supplement: S2 Fig — EMSA data reveals that the addition of the unlabeled 1xSPS15 and 2xCSL17 probes, but not the 2xCSL17mut probe results in decreased Su(H) binding to the labeled 2xCSL17 probe. 3.5nM labeled probe and 40nM Su(H) was used in indicated lanes. Three concentrations of each unlabeled competitor probe were tested with increases from 8.75nM to 140nM in four-fold steps. (TIF) [file pgen.1009039.s002.tif]

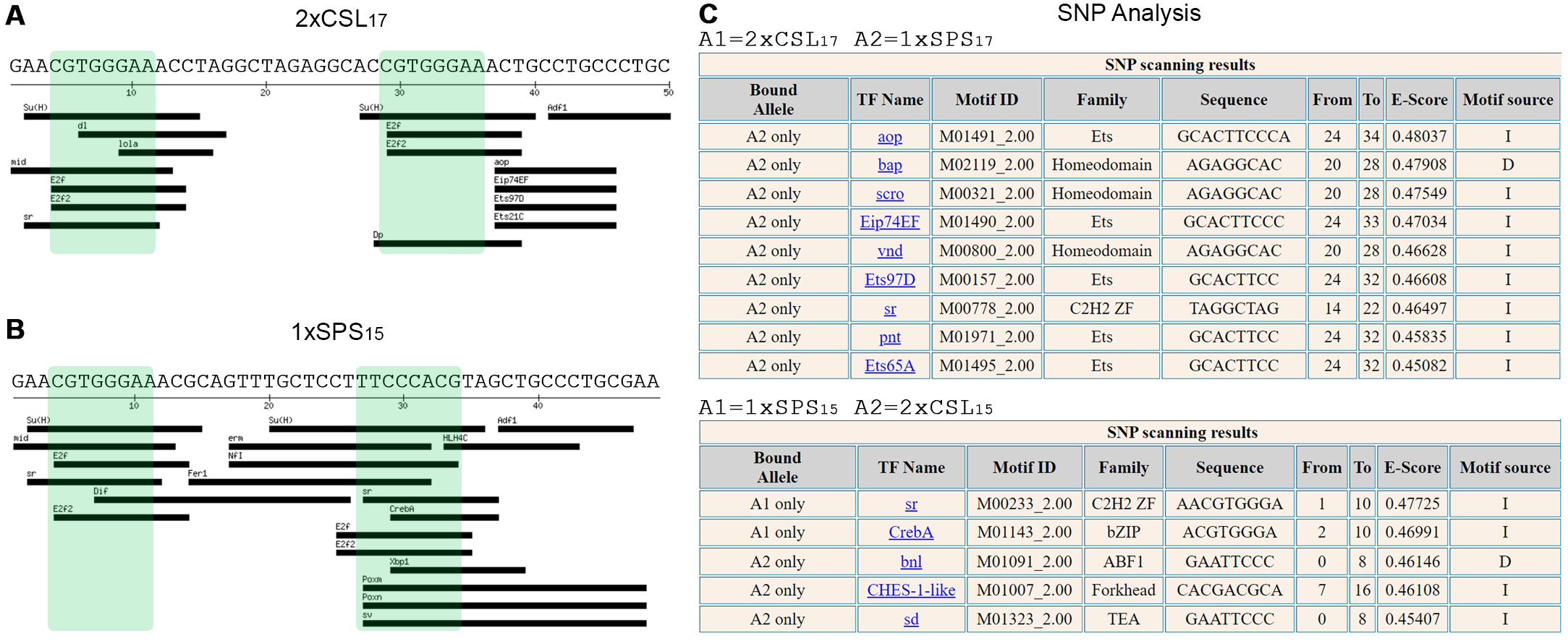

Supplement: S3 Fig — A-B. Transcription factor binding site prediction analysis of the 2xCSL17 and 1xSPS15 sequences for all known Drosophila TFs listed in the cis-BP database using a log-odds position weight matrix (PWM) score of 9 or higher. Note, the core 8bp sequence of the two Su(H) sites are highlighted in green and the other potential TF motifs are indicated by the black bars. C. SNP analysis between 2xCSL17 (A1 sequence, top) and 1xSPS17 (A2 sequence, top) and between 1xSPS15 (A1 sequence, bottom) and 2xCSL15 (A2 sequence, bottom) predicts acquired (A2 only) and/or lost (A1 only) transcription factor binding sites after inverting one of the Su(H) binding sites in 2xCSL17 or 1xSPS15. (TIF) [file pgen.1009039.s003.tif]

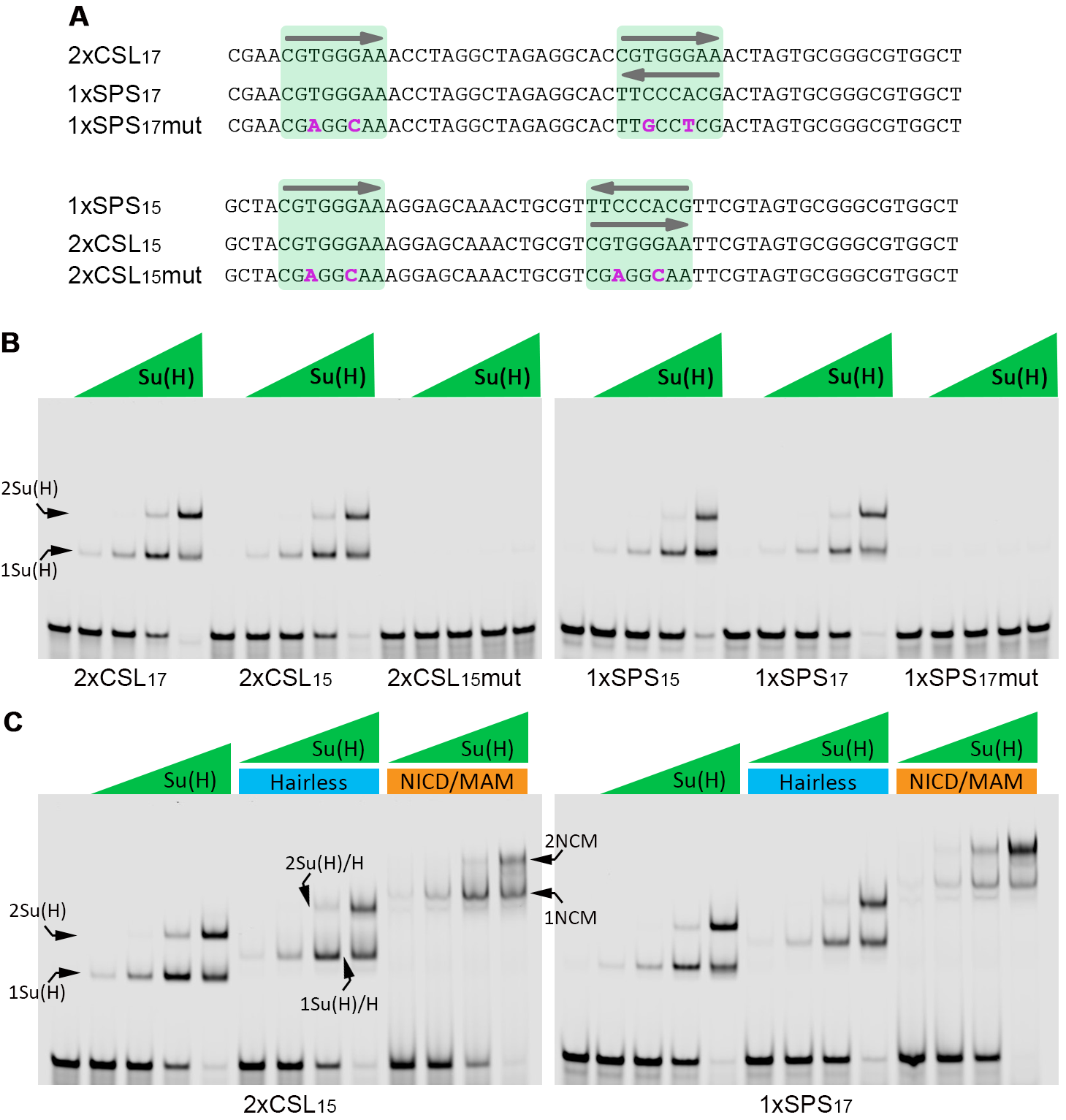

Supplement: S4 Fig — A. Sequences of the original 2xCSL17 and 1xSPS15 probes as well as the inverted 1xSPS17 and 2xCSL15 sequences with the orientation of each Su(H) site in each probe highlighted by an arrow. B. EMSAs reveal binding of purified Su(H) to the indicated probes. Su(H) concentration increases from 2.5nM to 160nM in 4-fold steps. C. EMSAs reveal binding of indicated purified proteins on 2xCSL15 and 1xSPS17 probes. Su(H) concentration increases from 2.5 to 160 nM in 4-fold steps and 2μM of either Hairless or NICD/MAM was used in the indicated lanes. (TIF) [file pgen.1009039.s004.tif]

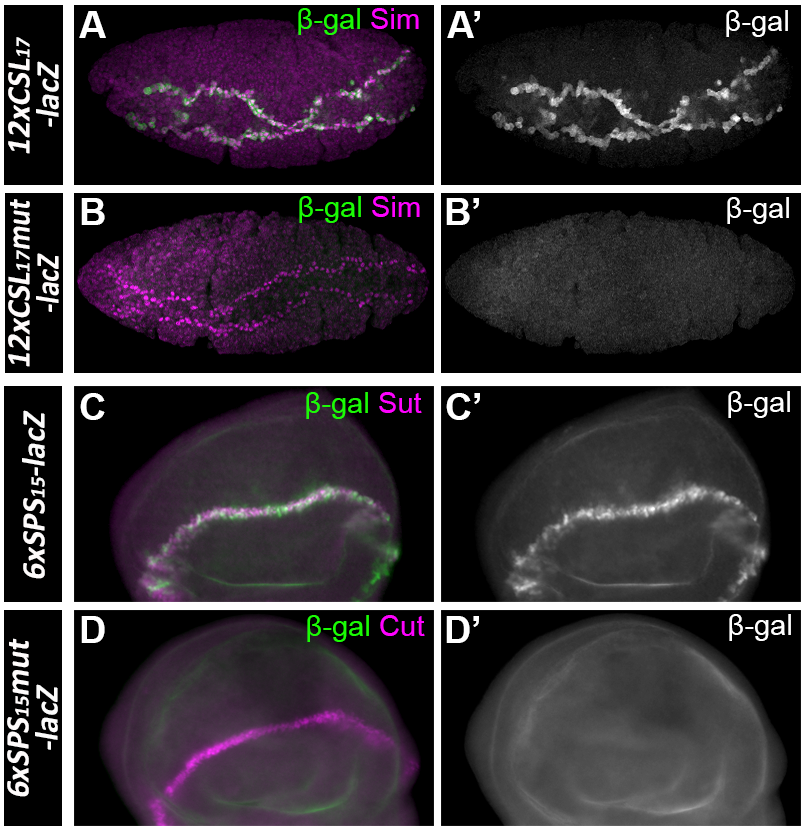

Supplement: S5 Fig — A-B. Stage 5 Drosophila embryos containing either the 12xCSL17-lacZ or the 12xCSLmut17-lacZ reporter were immunostained and imaged under identical conditions for β-gal (green, black and white in A’ and B’) and Sim (magenta). Note, the mesectoderm expression activity of the 12xCSL17-lacZ reporter is lost when the CSL binding sites were mutated. C-D. Larval wing discs containing either the 6xSPS15-lacZ or the 6xSPSmut15-lacZ reporter were immunostained and imaged under identical conditions for β-gal (green, black and white in C’ and D’) and Cut (magenta). Note, the wing margin cell expression activity of the 6xSPS15-lacZ reporter is lost when each SPS binding site was mutated. Each lacZ transgene was inserted into the ZH-86Fb locus. (TIF) [file pgen.1009039.s005.tif]

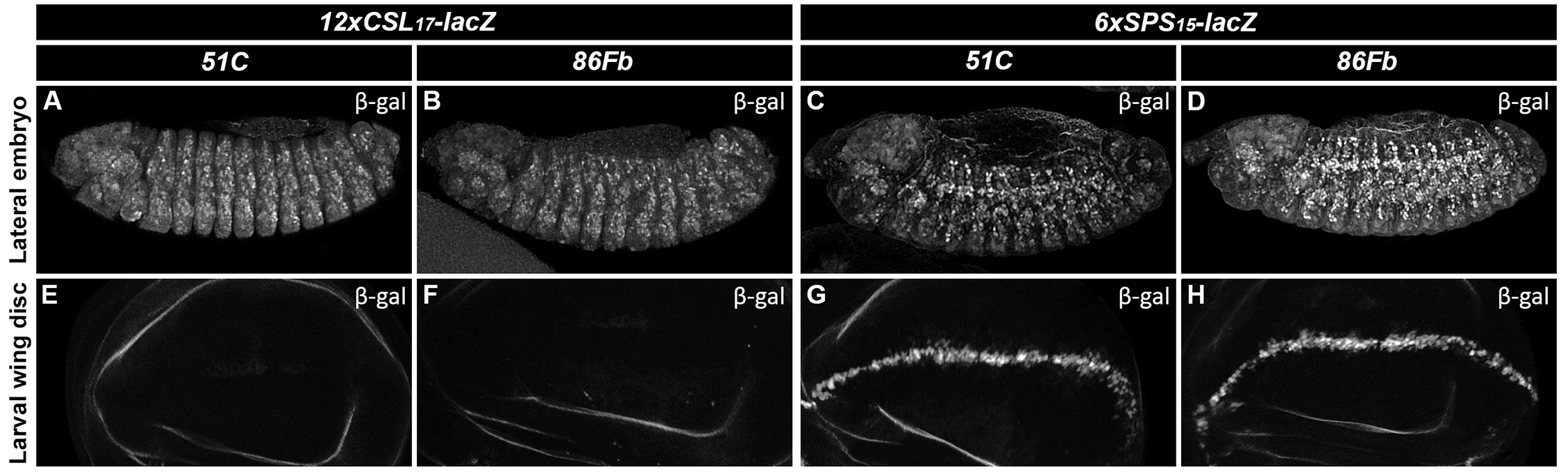

Supplement: S6 Fig — A-D. Stage 15 Drosophila embryos homozygous for either the 12xCSL17-lacZ (A-B) or 6xSPS15-lacZ (C-D) at the indicated genomic loci (51C or 86Fb) were immunostained with β-gal. Note, the similar expression patterns by both transgenes in each chromosomal location. E-H. Third instar larval wing imaginal discs homozygous for the indicated reporters were immunostained with β-gal. Note, only the 6xSPS15-lacZ reporter is active in the wing margin cells, whereas the 12xCSL17-lacZ fails to activate significant gene expression when inserted into either the 51C or 86Fb locus. (TIF) [file pgen.1009039.s006.tif]

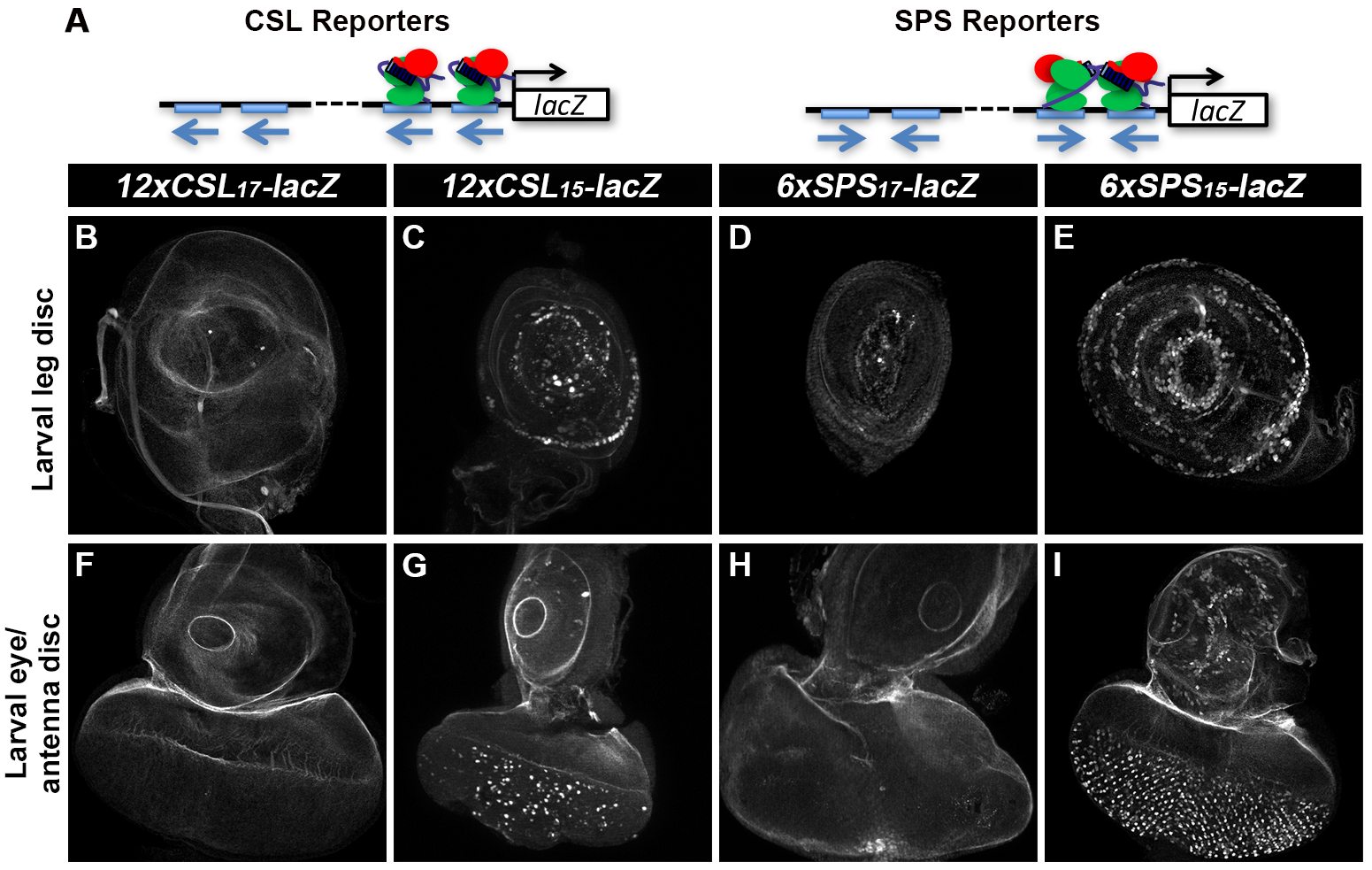

Supplement: S7 Fig — A. Schematics of the CSL and SPS reporter constructs with the orientation of each Su(H) binding site highlighted by an arrow. B-I. β-gal immunostaining of third instar larval imaginal discs reveals that the SPS and CSL reporters containing a 15bp spacer (C,E,G,I), but not the SPS and CSL reporters containing a 17bp spacer (B,D,F,H), are active in the expected pattern in larval leg discs (B-E) and larval eye-antenna discs (F-I). The 12xCSL17-lacZ and 6xSPS15-lacZ transgenes were inserted into the ZH-86Fb locus, and the 12xCSL15-lacZ and 6xSPS17-lacZ transgenes were inserted into the ZH-51C locus. (TIF) [file pgen.1009039.s007.tif]

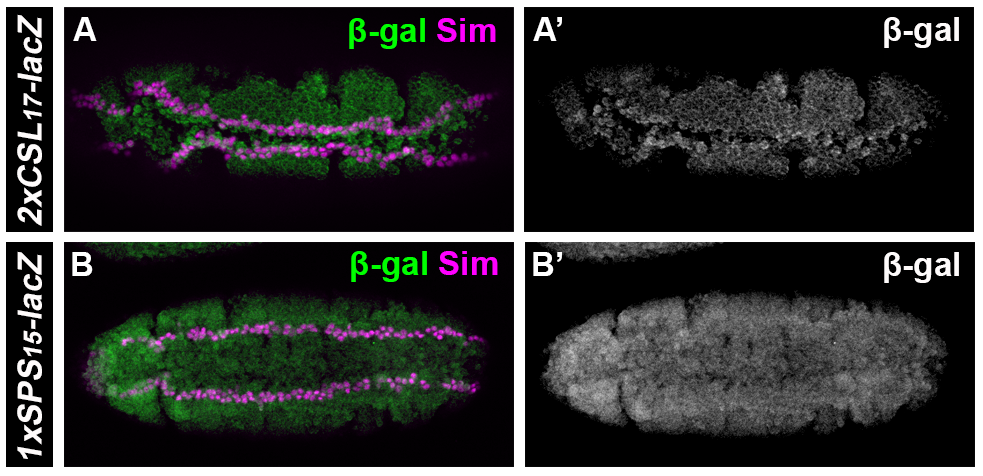

Supplement: S8 Fig — A-B. Stage 5 Drosophila embryos containing either the 2xCSL17-lacZ (A) or 1xSPS15-lacZ (B) reporter were immunostained and imaged under identical conditions for β-gal (green, black and white in A’ and B’) and Sim (magenta). Note, neither reporter activates in the mesectoderm. Both lacZ transgenes were inserted into the ZH-86Fb locus. (TIF) [file pgen.1009039.s008.tif]

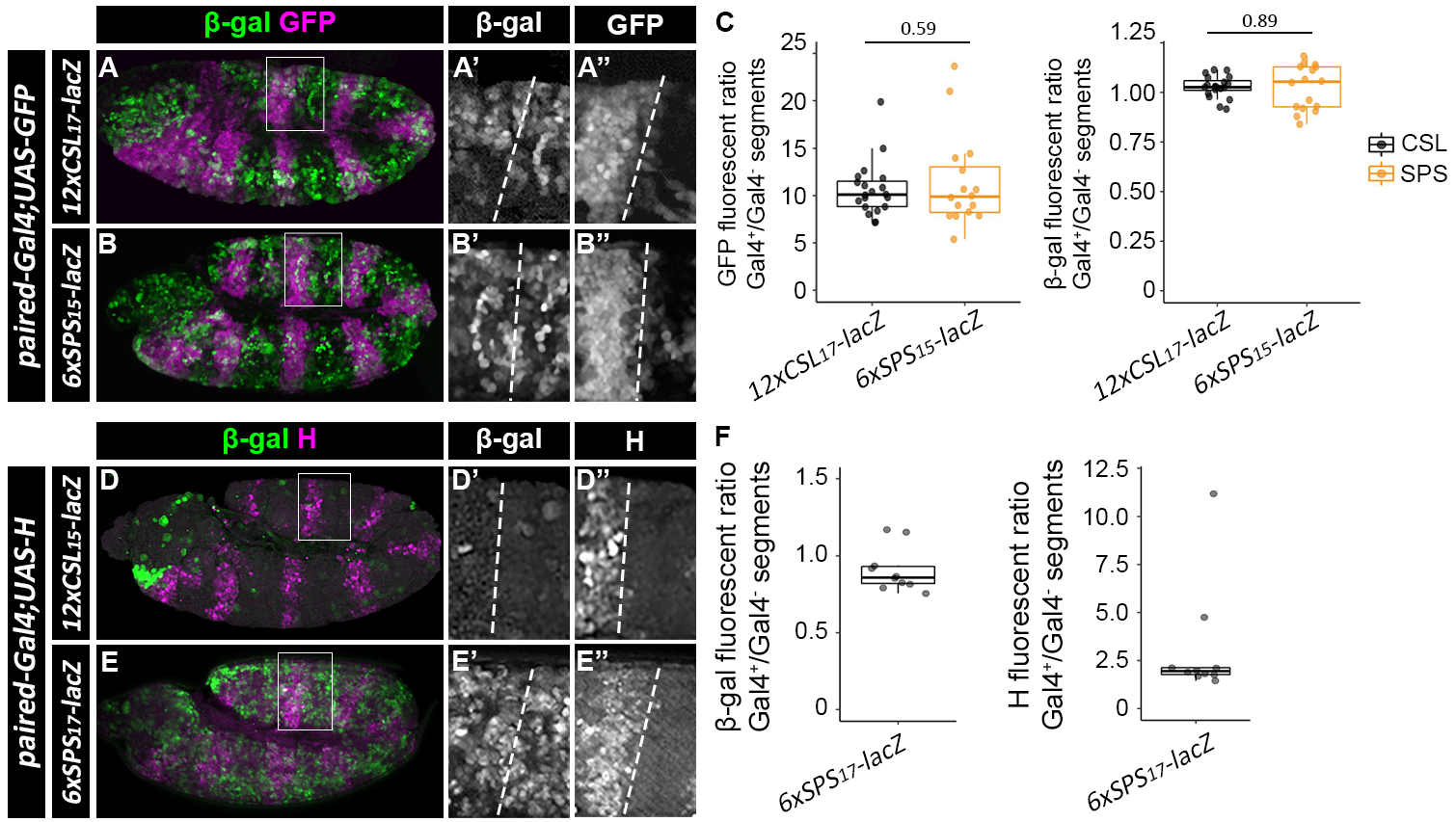

Supplement: S9 Fig — A-B. Lateral views of stage 11 paired-Gal4>UAS-GFP embryos containing either the 12xCSL17-lacZ (A) or 6xSPS15-lacZ (B) reporter inserted into the ZH-51C locus. Embryos were immunostained with β-gal (green) and GFP (magenta), and close-up views of the individual channels in black and white for the highlighted regions are shown in A’-B’ (β-gal) and A”-B” (GFP). C. Quantification of the ratio of β-gal and GFP in parasegments with ectopic GFP (paired-Gal4+) compared to control parasegments (paired-Gal4-). Each dot represents the mean measurement from an individual embryo containing either the 12xCSL17-lacZ or 6xSPS15-lacZ reporter. Sample size (n) is 20 for 12xCSL17-lacZ and 16 for 6xSPS15-lacZ. Box plots show the median, interquartile range, and 1.5 times interquartile range. One-way ANOVA was used to test significance. These data show that ectopic expression of GFP by paired-Gal4 does not dramatically impact either 12xCSL17-lacZ or 6xSPS15-lacZ activity. D-E. Lateral view of stage 11 paired-Gal4>UAS-Hairless embryos containing either the 12xCSL15-lacZ (D) or 6xSPS17-lacZ (E) reporter. Embryos were immunostained with β-gal (green) and Hairless (magenta), and close-up views of the individual channels in black and white for the highlighted regions are shown in D’-E’ (β-gal) and D”-E” (Hairless). Note, because the 12xCSL15-lacZ reporter is not active in the PrdG4 parasegments in the Drosophila embryo, we were unable to assess the impact of Hairless overexpression on this reporter. F. Quantification of ratios of β-gal and Hairless in parasegments with ectopic Hairless (paired-Gal4+) compared to control parasegments (paired-Gal4-). Each dot represents the mean measurement from an individual embryo containing 6xSPS17-lacZ reporter. Sample size (n) is 10. Box plots show the median, interquartile range, and 1.5 times interquartile range. Note, unlike the 12xCSL17-lacZ reporter expression that was strongly decreased by Hairless overexpression (see Fig 6), the 6xSPS17-lacZ repor [file pgen.1009039.s009.tif]

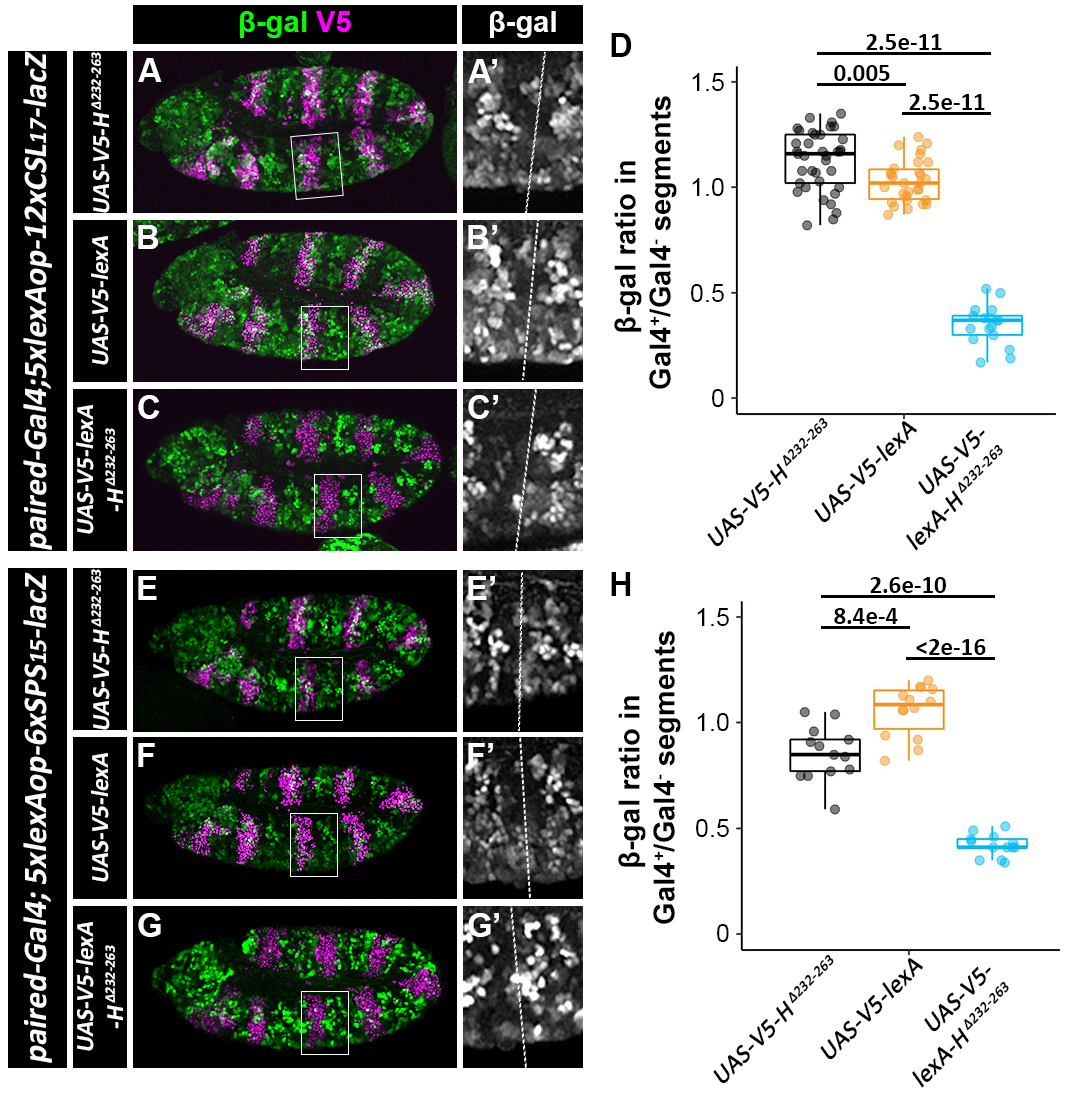

Supplement: S10 Fig — A-C. Stage 11 embryos of paired-Gal4;5xlexAop-12xCSL17-lacZ with either UAS-V5-HairlessΔ232-263(A), UAS-V5-lexA (B) or UAS-V5-lexA-HairlessΔ232-263(C) immunostained for β-gal (green) and the V5 epitope (magenta). A’-C’. Close-up views of β-gal intensity in black and white are shown in insets from A-C with the paired-Gal4-positive parasegment on the left and the paired-Gal4-negative parasegment on the right. D. Quantification of ratios of β-gal of paired-Gal4-positive over paired-Gal4-negative parasegments in paired-Gal4;5xlexAop-12xCSL17-lacZ flies with indicated UAS construct. Each dot represents the average measurement from an individual embryo. Sample sizes (n) are 37 for UAS-V5-HairlessΔ232–263, 30 for UAS-V5-lexA, and 17 for UAS-V5-lexA-HairlessΔ232–263. Box plots show the median, interquartile range, and 1.5 times interquartile range. One-way ANOVA with post-hoc Tukey HSD was used to test significance. E-G. Stage 11 embryos of paired-Gal4;5xlexAop-6xSPS15-lacZ with either UAS-V5-HairlessΔ232-263(E), UAS-V5-lexA (F) or UAS-V5-lexA-HairlessΔ232-263(G) immunostained for β-gal (green) and the V5 epitope (magenta). E’-G’. Close-up views of β-gal intensity in black and white are shown in insets from E-G. H. Quantification of ratios of β-gal of paired-Gal4-positive over paired-Gal4-negative parasegments in paired-Gal4;5xlexAop-6xSPS15-lacZ flies with indicated UAS construct. Each dot represents the average measurement from an individual embryo. Sample sizes (n) are 13 for UAS-V5-HairlessΔ232–263, 14 for UAS-V5-lexA, and 13 for UAS-V5-lexA-HairlessΔ232–263. Box plots show the median, interquartile range, and 1.5 times interquartile range. One-way ANOVA with post-hoc Dunnett’s T3 was used to test significance. A-G. All the lacZ transgenes were inserted into ZH-51C locus. (TIF) [file pgen.1009039.s010.tif]
