# Supplementary material for: Enhancers with cooperative Notch binding sites are more resistant to regulation by the Hairless co-repressor
Source: PLoS Genet. 2021 Sep 24;17(9):e1009039. doi: 10.1371/journal.pgen.1009039 (PMC8494340; doi:10.1371/journal.pgen.1009039)
Supplement: S1 Table — (DOCX) [file pgen.1009039.s012.docx]

**S1 Table**. EMSA probes.

| **Probe name** | **Sequence** |
| --- | --- |
| 2xCSL_17_ | CGAACGTGGGAAACCTAGGCTAGAGGCACCGTGGGAAACTAGTGCGGGCGTGGCT |
| 1xSPS_15_ | GCTACGTGGGAAAGGAGCAAACTGCGTTTCCCACGTTCGTAGTGCGGGCGTGGCT |
| 2xCSL_17_mut | CGAACGAGGCAAACCTAGGCTAGAGGCACCGAGGCAAACTAGTGCGGGCGTGGCT |
| 1xSPS_15_mut | GCTACGAGGCAAAGGAGCAAACTGCGTTTGCCTCGTTCGTAGTGCGGGCGTGGCT |
| 2xCSL_15_ | GCTACGTGGGAAAGGAGCAAACTGCGTCGTGGGAATTCGTAGTGCGGGCGTGGCT |
| 2xCSL_15_mut | GCTACGAGGCAAAGGAGCAAACTGCGTCGAGGCAATTCGTAGTGCGGGCGTGGCT |
| 1xSPS_17_ | CGAACGTGGGAAACCTAGGCTAGAGGCACTTCCCACGACTAGTGCGGGCGTGGCT |
| 1xSPS_17_mut | CGAACGAGGCAAACCTAGGCTAGAGGCACTTGCCTCGACTAGTGCGGGCGTGGCT |
| 5’IRDye-700 complementary_oligo | AGCCACGCCCGCACT |
| 5’IRDye-800 complementary_oligo | AGCCACGCCCGCACT |
