# Supplementary material for: Enhancers with cooperative Notch binding sites are more resistant to regulation by the Hairless co-repressor
Source: PLoS Genet. 2021 Sep 24;17(9):e1009039. doi: 10.1371/journal.pgen.1009039 (PMC8494340; doi:10.1371/journal.pgen.1009039)
Supplement: S2 Table — (DOCX) [file pgen.1009039.s013.docx]

**S2 Table**. Sequences used for molecular cloning.

| **Enhancer** | **Restriction Site** | **Sequence** |
| --- | --- | --- |
| 2xCSL_17_ | EcoRI/BglII | GAATTCGCCCTGCGAACGTGGGAAACCTAGGCTAGAGGCACCGTGGGAAACTGCCTAGATCT |
| 4xCSL_17_ | EcoRI/BglII | GAATTCGCCCTGCGAACGTGGGAAACCTAGGCTAGAGGCACCGTGGGAAACTGCCTGCCCTGCGAACCGTGGGAAACCTAGGCTAGAGGCACCGTGGGAAACTGCCTAGATCT |
| 8xCSL_17_ | EcoRI/Acc65I | GAATTCGCCCTGCGAACGTGGGAAACCTAGGCTAGAGGCACCGTGGGAAACTGCCTGCCCTGCGAACCGTGGGAAACCTAGGCTAGAGGCACCGTGGGAAACTGCCTAGATCTGCCCTGCGAACGTGGGAAACCTAGGCTAGAGGCACCGTGGGAAACTGCCTGCCCTGCGAACCGTGGGAAACCTAGGCTAGAGGCACCGTGGGAAACTGCCTGGTACC |
| 12xCSL_17_ | EcoRI/BglII | GAATTCGCCCTGCGAACGTGGGAAACCTAGGCTAGAGGCACCGTGGGAAACTGCCTGCCCTGCGAACGTGGGAAACCTAGGCTAGAGGCACCGTGGGAAACTGCCTGCCCTGCGAACGTGGGAAACCTAGGCTAGAGGCACCGTGGGAAACTGCCTGCCCTGCGAACGTGGGAAACCTAGGCTAGAGGCACCGTGGGAAACTGCCTGCCCTGCGAACGTGGGAAACCTAGGCTAGAGGCACCGTGGGAAACTGCCTGCCCTGCGAACGTGGGAAACCTAGGCTAGAGGCACCGTGGGAAACTGCCTAGATCT |
| 1xSPS_15_ | EcoRI/BglII | GAATTCAGCTACGTGGGAAAGGAGCAAACTGCGTTTCCCACGTTCGCAGGGCAGATCT |
| 2xSPS_15_ | EcoRI/BglII | GAATTCAGCTACGTGGGAAAGGAGCAAACTGCGTTTCCCACGTTCGCAGGGCAGCTACGTGGGAAAGGAGCAAACTGCGTTTCCCACGTTCGCAGGGCAGATCT |
| 4xSPS_15_ | EcoRI/Acc65I | GAATTCAGCTACGTGGGAAAGGAGCAAACTGCGTTTCCCACGTTCGCAGGGCAGCTACGTGGGAAAGGAGCAAACTGCGTTTCCCACGTTCGCAGGGCAGATCTAGCTACGTGGGAAAGGAGCAAACTGCGTTTCCCACGTTCGCAGGGCAGCTACGTGGGAAAGGAGCAAACTGCGTTTCCCACGTTCGCAGGGCGGTACC |
| 6xSPS_15_ | EcoRI/BglII | GAATTCAGCTACGTGGGAAAGGAGCAAACTGCGTTTCCCACGTTCGCAGGGCAGCTACGTGGGAAAGGAGCAAACTGCGTTTCCCACGTTCGCAGGGCAGCTACGTGGGAAAGGAGCAAACTGCGTTTCCCACGTTCGCAGGGCAGCTACGTGGGAAAGGAGCAAACTGCGTTTCCCACGTTCGCAGGGCAGCTACGTGGGAAAGGAGCAAACTGCGTTTCCCACGTTCGCAGGGCAGCTACGTGGGAAAGGAGCAAACTGCGTTTCCCACGTTCGCAGG GCAGATCT |
| 12xCSL_17_mut | EcoRI/BglII | GAATTCGCCCTGCGAACGAGGCAAACCTAGGCTAGAGGCACCGAGGCAAACTGCCTGCCCTGCGAACGAGGCAAACCTAGGCTAGAGGCACCGAGGCAAACTGCCTGCCCTGCGAACGAGGCAAACCTAGGCTAGAGGCACCGAGGCAAACTGCCTGCCCTGCGAACGAGGCAAACCTAGGCTAGAGGCACCGAGGCAAACTGCCTGCCCTGCGAACGAGGCAAACCTAGGCTAGAGGCACCGAGGCAAACTGCCTGCCCTGCGAACGAGGCAAACCTAGGCTAGAGGCACCGAGGCAAACTGCCTAGATCT |
| 6xSPS_15_mut | EcoRI/BglII | GAATTCAGCTACGAGGCAAAGGAGCAAACTGCGTTTGCCTCGTTCGCAGGGCAGCTACGAGGCAAAGGAGCAAACTGCGTTTGCCTCGTTCGCAGGGCAGCTACGAGGCAAAGGAGCAAACTGCGTTTGCCTCGTTCGCAGGGCAGCTACGAGGCAAAGGAGCAAACTGCGTTTGCCTCGTTCGCAGGGCAGCTACGAGGCAAAGGAGCAAACTGCGTTTGCCTCGTTCGCAGGGCAGCTACGAGGCAAAGGAGCAAACTGCGTTTGCCTCGTTCGCAG GGCAGATCT |
| 12xCSL_15_ | HindIII/BglII | AAGCTTAGCTACGTGGGAAAGGAGCAAACTGCGTCGTGGGAATTCGCAGGGCAGCTACGTGGGAAAGGAGCAAACTGCGTCGTGGGAATTCGCAGGGCAGCTACGTGGGAAAGGAGCAAACTGCGTCGTGGGAATTCGCAGGGCAGCTACGTGGGAAAGGAGCAAACTGCGTCGTGGGAATTCGCAGGGCAGCTACGTGGGAAAGGAGCAAACTGCGTCGTGGGAATTCGCAGGGCAGCTACGTGGGAAAGGAGCAAACTGCGTCGTGGGAATTCGCAGGGCAGATCT |
| 6xSPS_17_ | EcoRI/BglII | GAATTCGCCCTGCGAACGTGGGAAACCTAGGCTAGAGGCACTTCCCACGACTGCCTGCCCTGCGAACGTGGGAAACCTAGGCTAGAGGCACTTCCCACGACTGCCTGCCCTGCGAACGTGGGAAACCTAGGCTAGAGGCACTTCCCACGACTGCCTGCCCTGCGAACGTGGGAAACCTAGGCTAGAGGCACTTCCCACGACTGCCTGCCCTGCGAACGTGGGAAACCTAGGCTAGAGGCACTTCCCACGACTGCCTGCCCTGCGAACGTGGGAAACCTAGGCTAGAGGCACTTCCCACGACTGCCTAGATCT |
| 5xlexAop | HindIII/EcoRI | AAGCTTTCTGTATATATATACAGACGCAGTTTGCTCCTCTGTATATATATACAGTAGCTGCCCTGCGATCTGTATATATATACAGACGCAGTTTGCTCCTCTGTATATATATACAGTAGCTGCCCTGCGATCTGTATATATATACAGCCTAGGGTAGCATGCGTAACCGGTGTAGAATTC |
| LexA-DBD | NdeI/BglII | CATATGCCACCCAAGAAGAAGCGAAAAGTAGAAGATCCAATGAAGGCTCTCACGGCCCGACAACAGGAAGTTTTTGATTTGATACGGGATCATATATCCCAAACGGGTATGCCTCCGACCCGCGCAGAGATAGCACAGCGACTGGGCTTTCGATCGCCTAACGCCGCGGAGGAGCACTTGAAGGCACTGGCCCGCAAGGGTGTCATTGAAATCGTGTCCGGTGCGAGCCGCGGAATCCGGCTGTTGCAGGAGGAGGAAGAGGGCCTGCCACTGGTGGGACGCGTGGCCGCTGGCGAGCCGCTGCTGGCCCAGCAACACATAGAGGGACACTATCAGGTGGACCCCTCCTTGTTTAAGCCAAATGCTGATTTCCTGTTGCGGGTGTCGGGAATGTCCATGAAGGACATCGGTATTATGGATGGTGACCTCCTCGCCGTCCATAAGACACAGGATGTCAGGAACGGCCAGGTAGTCGTTGCCAGGATAGACGATGAGGTCACTGTGAAACGTCTCAAGAAGCAAGGCAATAAGGTCGAGCTGCTGCCGGAGAATAGCGAGTTCAAGCCGATCGTGGTGGATCTGCGACAGCAGTCCTTTACTATCGAGGGCTTGGCCGTGGGTGTGATCCGCAACGGAGATTGGCTGGGCTCCGGCTCAGATCT |
| Hairless∆232-263 | BglII/KpnI | AGATCTGATGGCCCTGCTTAATGACGTCACAAGCGTAGCAGAGTGCAACAGACAGACAACAATGACCGATGAGCATAAAAGTAACATTAACAGTAACAGCAGTCACTCCAGCAACAACAACAACAACGGCAGCAGCAGCAATAACGACAACAACAGCAACGACGACGCAGCAAGTAGCAGCAACAGCAAAAACAACAACACCAGCAACGAGAGCAGCCACAGCAACAACAATACTAGTAGCATAATTGCAGAGGCGGCCGCCAAGTTTCTACTGAAAAATGGCCTAAACGGCAGTAGCAGCACCAGCTACCCCCCTCTGCCACCGCCTCTGCCCGCCAACTTAAGCAGGACGACCACGCCCACGACAACGACAACGCCCTCATCCTCCAGCTCCACCGCCTCAAATGGCTTTTTGCCGCATGCCAAGACGCCCAAAAGTAGTAGCATTATGGCTGCGTCCGCCGCAGTGGCAGCCAGCGTCGTTGGAGCTACTGCGTCCAAGCCCACCATCGATGTCCTGGGGGGCGTCCTGGACTACAGTTCCTTGGGCGGAGCTGCAACAGGCTCACTGCCCACCACTGCAGTAGTAGCGGCGGCAGCGGGAACAGCGAAGATCGGCAAGGGAAGCAACTCCGGCGGAAGCTTTGATATGGGCAGGACACCAATATCGACGCACGGCAACAACAGCTGGGGCGGCTACAAGACCTTCCGCCCTCCATCGGCGGCCACCTCCGCAACTGTGACCCCAACGTCGGCGGTGACCACAGCGTATCCAAAGAACGAGAATTCCACATCGCTGAGCTTTTCGGACGACAACAGCTCGATACAATCCTCTCCTTGGCAGCGAGACCAGCCCTGGAAACAGTCCCGACCCAGGCGCGGCATATCTAAGGAGCTGTCGCTCTTCTTCCACCGCCCCAGGAACAGTACGCTTGGCCGAGCTGCTCTCCGGACAGCCGCTCGCAAACGACGGCGCCCCCACGAGCCGCTTACCACCAGCGAGGATCAGCAGCCCATTTTTGCGACGGCAATCAAGGCGGAAAATGGAGACGATACTCTTAAAGCAGAAGCTGCAGAGGCCGTTGAAATTGAAAATGTTGCTGTGGCGGACACAACCACAAATGAGATTAAAATTGAAAAACCGGACACGATCAAAGGCGAGGATGATGCTGAACGGCTCGAAAAGGAGCCGAAGAAGGCGGTTAGCGATGATAGCGAGTCAAAAGAAGCATCGCCCGGTCAGCAAGTGGAACCACAACCAAAAGATGAGACTGTTGATGTTGAGATGAAGATGAATACGAGCGAGGATGAGGAACCCATGACAGAGCTGCCCAGAATCACGAATGCCGTAAATGGTGATCTAAACGGCGATCTAAAGGCGAGCATTGGGAAACCAAAATCCAAGCCGAAGCCAAAAGCCAAGCTCAGCAGCATCATTCAGAAACTCATCGATAGCGTACCAGCACGGCTTGAGCAAATGTCGAAGACATCAGCTGTGATCGCATCGACAACGACGTCTTCAGATCGCATTGGTGGCGGTCTAAGTCACGCCTTGACGCACAAAGTTTCTCCACCCTCTTCTGCGACAGCAGCCGGACGACTAGTCGAGTACCACACCCAGCACGTGTCGCCCAGGAAAAGAATCCTGCGCGAGTTCGAAAAGGTGTCGCTAGAGGACAACGGATGCGTAAACAACGGCAGCGGTGGAGCTAGTAGCGGTGGTGCTGGAGGAAAACGGAGTCGAGCAAAGGGAACTTCGACATCGTCTCCGGCTGGCAAGGCGTCACCAATGAACTTGGCGCCACCCCAAGGAAAGCCAAGCCCCAGTCCCGGCTCCAGCTCATCCAGCACTTCGCCAGCGACCTTGTCAACGCAGCCAACGCGGCTCAACAGCTCTTACAGTATCCACTCCCTGCTAGGTGGGAGCAGTGGCAGCGGTAGCTCATCCTTCTCCTCCTCTGGCAAGAAGTGCGGCGATCACCCGGCAGCTATTATCAGCAATGTGCACCATCCACAGCACTCAATGTACCAACCCAGTTCCTCGAGCTATCCACGCGCCCTGCTCACCTCGCCAAAGTCGCCCGATGTGAGTGGCAGCAATGGCGGGGGCGGAAAATCGCCCTCGCATACAGGAACCAAGAAGCGTTCGCCACCGTACTCGGCGGGATCACCCGTAGACTATGGCCACTCCTTCTACAGGGATCCCTATGCGGGAGCAGGTCGTCCTTCCACATCGGGCTCAGCATCGCAGGACCTGTCGCCACCGCGCTCTTCCCCAGCATCGCCAGCCACGACGCCGCGTACTGTGCCCAAAAAGACTGCATCGATCCGACGCGAGTTCGCTTCACCGTCGGCCAGCAGCAGTAGCTGTCCCTCGCCCGGCGACCGGAGTGCATCGCCCCCGGAACGGCGGCACATGCAGCAGCAGCCGCACCTACAGCGTAGCTCGCCGCTGCACTACTATATGTACCCGCCACCGCCCCAGGTGAACGGGAACGGCTCGGCCGGAAGTCCGACCTCGGCGCCGCCCACGTCGAACAGCAGTGCAGCTGCAGTAGCGGCGGCAGCAGCGGCCGCAGCCGCATACATTCCCTCGCCTTCGATATACAACCCGTACATATCCACACTGGCGGCGTTGAGGCACAATCCGCTGTGGATGCACCACTATCAGACAGGAGCGTCGCCCCTGCTGTCGCCACATCCACAACCCGGTGGCTCAGCGGCCGCCGCTGCTGCAGCTGCTGCTGCGAGATTATCGCCCCAATCGGCCTATCACGCGTTCGCGTATAACGGAGTGGGAGCGGCTGTTGCCGCTGCAGCAGCTGCGGCAGCCTTTGGACAACCGGCGCCCAGTCCCCACACGCATCCGCACTTGGCCCATCCGCACCAGCATCCGCACCCGGCTGCACTGACCACCCACCACTCTCCCGCTCACCTGGCCACGCCAAAACTGACTGATAGTAGTACCGACCAAATGTCTGCAACGTCCAGTCATCGCACAGCCTCCACTTCGCCGAGCAGCTCGAGCGCATCGGCCTCCTCCTCGGCGGCCACTTCGGGCGCCAGCTCCTCCGCAATGTTTCATACTAGTAGTCTAAGGAATGAACAAAGTTCAGACTTACCACTGAATCTGTCAAAGCACTGAGGTACC |
